# Incident type 2 diabetes and its risk factors in men and women aged 40–60 years from four sub-Saharan African countries: results from the AWI-Gen study

**DOI:** 10.1016/S2214-109X(24)00520-5

**Published:** 2025-03

**Authors:** Raylton P Chikwati, Nigel J Crowther, Michèle Ramsay, Lisa K Micklesfield, Shane A Norris, Kagiso P Seakamela, Engelbert A Nonterah, Godfred Agongo, Shukri F Mohamed, Isaac Kisiangani, Palwende R Boua, Alisha N Wade

**Affiliations:** Sydney Brenner Institute for Molecular Bioscience, Faculty of Health Sciences, University of the Witwatersrand, Johannesburg, South Africa; Department of Chemical Pathology, National Health Laboratory Service, Faculty of Health Sciences, University of the Witwatersrand, Johannesburg, South Africa; Sydney Brenner Institute for Molecular Bioscience, Faculty of Health Sciences, University of the Witwatersrand, Johannesburg, South Africa; SAMRC/Wits Developmental Pathways for Health Research Unit, Department of Paediatrics, School of Clinical Medicine, Faculty of Health Sciences, University of the Witwatersrand, Johannesburg, South Africa; SAMRC/Wits Developmental Pathways for Health Research Unit, Department of Paediatrics, School of Clinical Medicine, Faculty of Health Sciences, University of the Witwatersrand, Johannesburg, South Africa, School of Human Development and Health, University of Southampton, Southampton, UK; Dikgale Mamabolo Mothiba (DIMAMO) Population Health Research Centre, University of Limpopo, Polokwane, South Africa; Navrongo Health Research Centre, Ghana Health Service, Navrongo, Ghana, Department of Epidemiology, School of Public Health, C K Tedam University of Technology and Applied Sciences, Navrongo, Ghana; Navrongo Health Research Centre, Ghana Health Service, Navrongo, Ghana, Department of Biochemistry and Forensic Sciences, School of Chemical and Biochemical Sciences, C K Tedam University of Technology and Applied Sciences, Navrongo, Ghana; Chronic Disease Management Unit, African Population and Health Research Centre, Nairobi, Kenya; Chronic Disease Management Unit, African Population and Health Research Centre, Nairobi, Kenya;; Sydney Brenner Institute for Molecular Bioscience, Faculty of Health Sciences, University of the Witwatersrand, Johannesburg, South Africa; Clinical Research Unit of Nanoro, Institut de Recherche en Sciences de la Santé, Nanoro, Burkina Faso; Medical Research Unit The Gambia at London School of Hygiene & Tropical Medicine, Fajara, The Gambia; Research in Metabolism and Endocrinology, Department of Internal Medicine, School of Clinical Medicine, Faculty of Health Sciences, University of the Witwatersrand, Johannesburg, South Africa; MRC/Wits Rural Public Health and Health Transitions Research Unit, School of Public Health, Faculty of Health Sciences, University of the Witwatersrand, Johannesburg, South Africa; Division of Endocrinology, Diabetes, and Metabolism, Perelman School of Medicine, University of Pennsylvania, Philadelphia, PA, USA

## Abstract

**Background:**

The incidence of type 2 diabetes in sub-Saharan Africa is expected to increase, but few longitudinal studies have characterised its risk factors. This study aimed to determine the incidence of type 2 diabetes over 33 481 person-years and identify its principal risk factors in middle-aged adults (ie, those aged 40–60 years) from four sub-Saharan African countries.

**Methods:**

Longitudinal data were available from 6553 participants aged 40–60 years at baseline from study centres in South Africa, Kenya, Ghana, and Burkina Faso. Sociodemographic, behavioural, clinical, and biochemical data were collected at baseline and after an interval of 5–6 years. The prevalence of type 2 diabetes was determined at each timepoint and diabetes incidence was calculated. A two-stage individual participant data meta-analysis was used to identify baseline risk factors for incident diabetes.

**Findings:**

The overall incidence of type 2 diabetes was 14∙6 (95% CI 13∙4–16∙0) cases per 1000 person-years. The incidence was highest in South Africa with 21∙8 (19∙5–24∙4) cases per 1000 person-years, and lowest in west Africa with 5∙5 (4∙4–6∙9) cases per 1000 person-years. Baseline glucose (adjusted odds ratio 1∙37; 95% CI 1∙16–1∙42), being male (1∙32; 1·12–1·54), family history of type 2 diabetes (1∙22; 1∙01–1∙46), unemployment (1∙19; 1∙03–1∙37), hypertension (1∙21; 1∙01–1∙45), BMI (1∙03; 1∙02–1∙04), and waist circumference (1∙02; 1∙01–1∙03), were associated with a higher risk of incident type 2 diabetes, while adequate baseline physical activity (0∙87; 0∙76–1∙00) was associated with lower risk.

**Interpretation:**

The high incidence of type 2 diabetes in this middle-aged sub-Saharan Africa population is influenced by several modifiable risk factors that should inform interventions to mitigate the disease burden.

## Introduction

Type 2 diabetes has emerged as a major threat to health-care systems and livelihoods in sub-Saharan Africa with the prevalence increasing from 4 million cases in 1980 to 23∙6 million cases in 2021, marking a 490% increase.^1^ Without effective interventions, the prevalence of type 2 diabetes is projected to double to 54∙9 million (95% CI 34∙8–69∙7) by 2045.^1^ However, type 2 diabetes does not affect all populations in sub-Saharan Africa equally, with population-based studies showing wide geographical and urban–rural variations in prevalence, with estimates ranging from 1∙4% in rural Uganda in east Africa to 17∙9% in urban Senegal in west Africa.^2^ Furthermore, the prevalence of type 2 diabetes also varies by age, with the highest prevalence in economically active individuals aged 40 years and older.^3^ In a multicountry cross-sectional study from sub-Saharan Africa, the prevalence of type 2 diabetes in individuals aged between 40 years and 60 years was 5.5% (95% CI 4∙4–6∙5).^4^

Middle-aged adults (ie, those aged 40–60 years) are at high risk for diabetes due to the confluence of lifestyle and physiological factors that contribute to the development of type 2 diabetes.^5^ The lifestyle factors include reduced physical activity and the physiological factors include increased adiposity and insulin resistance and hormonal changes.^5^ Sex differences have also been shown to play a major role in the development of type 2 diabetes.^6^ In 2021, approximately 17∙7 million more men than women were living with diabetes across the world.^3^ Evidence also shows that women bear a greater burden of type 2 diabetes risk factors at the time of diabetes diagnosis, particularly obesity.^6^ Menopause has been independently associated with obesity and increased glucose levels.^7^ Women with type 2 diabetes have been shown to have a higher relative risk of cardiovascular disease and mortality than males, although males still have a higher absolute risk.^6^ Thus, the risk factors for developing diabetes can vary by sex and therefore understanding how sex and other risk factors influence diabetes in sub-Saharan Africa can inform public health initiatives.

Most studies on type 2 diabetes in sub-Saharan Africa have been cross-sectional, and prospective data on the risk factors of incident diabetes are limited. Studies of diabetes incidence have been restricted to single centres, limiting their generalisability,^8–11^ because there is considerable variation across sub-Saharan Africa in several factors that could influence incident diabetes such as demographic and anthropometric variables, social determinants of health, including urbanisation, and the prevalence of HIV and tuberculosis. Longitudinal, multinational studies are needed in sub-Saharan Africa to quantify the incidence of diabetes, identify major risk factors, and determine whether these differ across contexts.

In this study, we aimed to investigate the incidence of type 2 diabetes and its associated risk factors in a cohort of individuals from south, east, and west sub-Saharan Africa, aged between 40 years and 60 years at cohort entry.

## Methods

### Study design

The Africa-Wits International Network for the Demographic Evaluation of Populations and Their Health (INDEPTH) Partnership for Genomic studies (AWI-Gen) is a longitudinal multicentre study of 10 702 participants at baseline, conducted in six sub-Saharan African centres: three in South Africa (Agincourt, DIMAMO [ formerly known as Dikgale], and Soweto), one in east Africa (Nairobi, Kenya), and two in West Africa (Navrongo, Ghana and Nanoro, Burkina Faso).^12^ AWI-Gen was designed to study genomic and environmental determinants of cardiometabolic diseases in sub-Saharan Africa.^12^ Participants were recruited between 2013 and 2017 and followed up between 2018 and 2022.

### Participant recruitment and sampling

Participants were eligible for recruitment if they were aged 40–60 years. Pregnant women and individuals who could not complete the prescribed study procedures were excluded. Participants were classified as female or male depending on their response to a question regarding sex assigned at birth. Details of the recruitment strategy have been described previously.^12^

Ethical approval was obtained from the Human Research Ethics Committee (Medical) of the University of the Witwatersrand (M121029 and M170880). Local ethics approval was obtained from the respective in-country ethical bodies. All participants provided written informed consent following community engagement and individual information sharing.

Participants were selected using random sampling based on existing population sampling frames from each study centre. In Agincourt, DIMAMO, Nairobi, Nanoro, and Navrongo, participants were recruited from residents within Health and Demographic Surveillance Systems. These systems were research infrastructures designed to collect longitudinal data on health and demographic changes within populations at each study centre. In Soweto, women were recruited from a pre-existing study on menopause,^13^ whereas men were selected from the general population. Further details are provided in the appendix (p 2).

### Data collection and variable descriptions

Data collection methods for AWI-Gen have been described previously,^12^ with the same procedures used at both baseline and follow-up visits. A standardised questionnaire was used to collect sociodemographic and personal and family medical history data. Trained staff used standardised protocols to measure waist and hip circumference, standing height, weight, and systolic and diastolic blood pressure and collect blood specimens.^12^ All biochemical assays were conducted at the same laboratory. Further details are presented in the appendix (p 3).

Type 2 diabetes was defined as a fasting plasma glucose concentration of 7∙0 mmol/L or more, a random plasma glucose concentration of 11∙1 mmol/L or more, or a previous diagnosis by a health-care professional.^14^ Impaired fasting glucose was defined as fasting glucose levels between 6∙1 mmol/L and 6∙9 mmol/L.^15^ Hypertension was defined as a systolic blood pressure of 140 mm Hg or more or a diastolic blood pressure of 90 mm Hg or more;^16^ or a previous diagnosis by a healthcare professional.

### Outcomes

The primary outcome was the incidence of type 2 diabetes. Secondary outcomes included the baseline risk factors, including sociodemographic, behavioural, anthropometric, and biochemical variables associated with incident type 2 diabetes and the change in type 2 diabetes prevalence between baseline and follow-up.

### Statistical analysis

We present continuous parametric data as means (SD), non-parametric data as medians with interquartile ranges (IQR), and categorical data as counts with percentages and confidence intervals (95% CI). Survivorship bias was assessed by comparing baseline differences between retained and lost participants. Bonferroni correction was applied to adjust for multiple testing.

We report the incidence of type 2 diabetes as cases per 1000 person-years and as a crude percentage of follow-up participants, excluding baseline type 2 diabetes cases. Incidence and prevalence were calculated for each study centre. Age-adjusted prevalence (95% CI) was calculated using the sub-Saharan Africa population distribution from the UN as the reference.^17^ We conducted Poisson regression analyses to explore the influence of sex and regional location on diabetes incidence.

We compared baseline anthropometric and biochemical variables between study participants who developed type 2 diabetes during the follow-up period and those who did not. Additionally, we determined the absolute change in these variables between the two visits and compared the changes between those who did and did not develop diabetes. Random blood glucose measurements were excluded from baseline and follow-up glucose difference calculations.

Logistic regression was used to determine the associations between baseline risk factors and incident type 2 diabetes (ie, occurrence or non-occurrence of diabetes). Risk factors were selected based on biological plausibility. Effect sizes for age, BMI, waist circumference, glucose and triglyceride levels, homoeostatic model assessment for insulin resistance, sex, employment status, physical activity, smoking, family history of diabetes, and hypertension were obtained from multivariable logistic regression models at each centre and pooled in two-stage individual meta-analyses. We also performed similar analyses in a combined sample of the four centres in east Africa and South Africa where HIV and tuberculosis were prevalent. In all meta-analyses, effect sizes were derived using random-effects models with inverse-variance weighting. Heterogeneity between study centres was assessed using the *I*^²^ and Tau (τ^²^) indices and Cochran’s Q statistic. Further details are described in the appendix (p 5). Pairwise deletion was applied for missing variables. The level of statistical significance was set at a two-tailed p*<*0∙05. All statistical analyses and visualisations were performed using Stata (version 18.0) and RStudio 4.0 (RStudio Team, PBC, Boston, MA, USA).

### Role of the funding source

The funders of the study had no role in the study design, data collection, data analyses, data interpretation, or writing of the report.

## Results

Of 10 702 individuals, we excluded 106 (1∙0%) who reported different dates of birth at baseline and at follow-up, and whose reported date of birth at follow up excluded them from the 40–60 year age group. Of the 10 596 participants recruited at baseline, 6553 (61∙8%) were retained at the follow-up visit (appendix p 29). More females were retained in the cohort than males (appendix pp 6–7). BMI and waist and hip circumference were higher in those who were retained in the cohort than those lost to follow-up, but these differences were not considered clinically significant. The baseline type 2 diabetes prevalence was higher at the South African and Kenyan centres than at both of the west African centres (appendix pp 8–9). The incidence of type 2 diabetes was significantly higher in peri-urban centres (23∙9, 95% CI 20∙5–27∙9) compared with urban centres (19∙7, 17∙4–22∙4), and both peri-urban and urban centres had higher incidences than rural centres (5∙5, 4∙4–6∙9; p<0∙0001).

Median duration of follow-up was 6 years (IQR 5–6). Overall type 2 diabetes incidence was 14∙6 (95% CI 13∙4–16∙0) cases per 1000 person-years. Incidence was highest in South Africa with 21∙8 (19∙5–24∙4) cases per 1000 person-years, and lowest in west Africa with 5∙5 (4∙4–6∙9) cases per 1000 person-years (table 1; appendix p 10). Overall, there were no differences in type 2 diabetes incidence between males and females, but females had a higher incidence in South Africa, and males had a higher incidence in west Africa (table 1; appendix p 10). Furthermore, males from South Africa and east Africa had higher incidence compared with those from west Africa (appendix p 10). A similar association was observed in females, but the magnitude of the difference was greater (appendix p 10).

**Figure 1:**
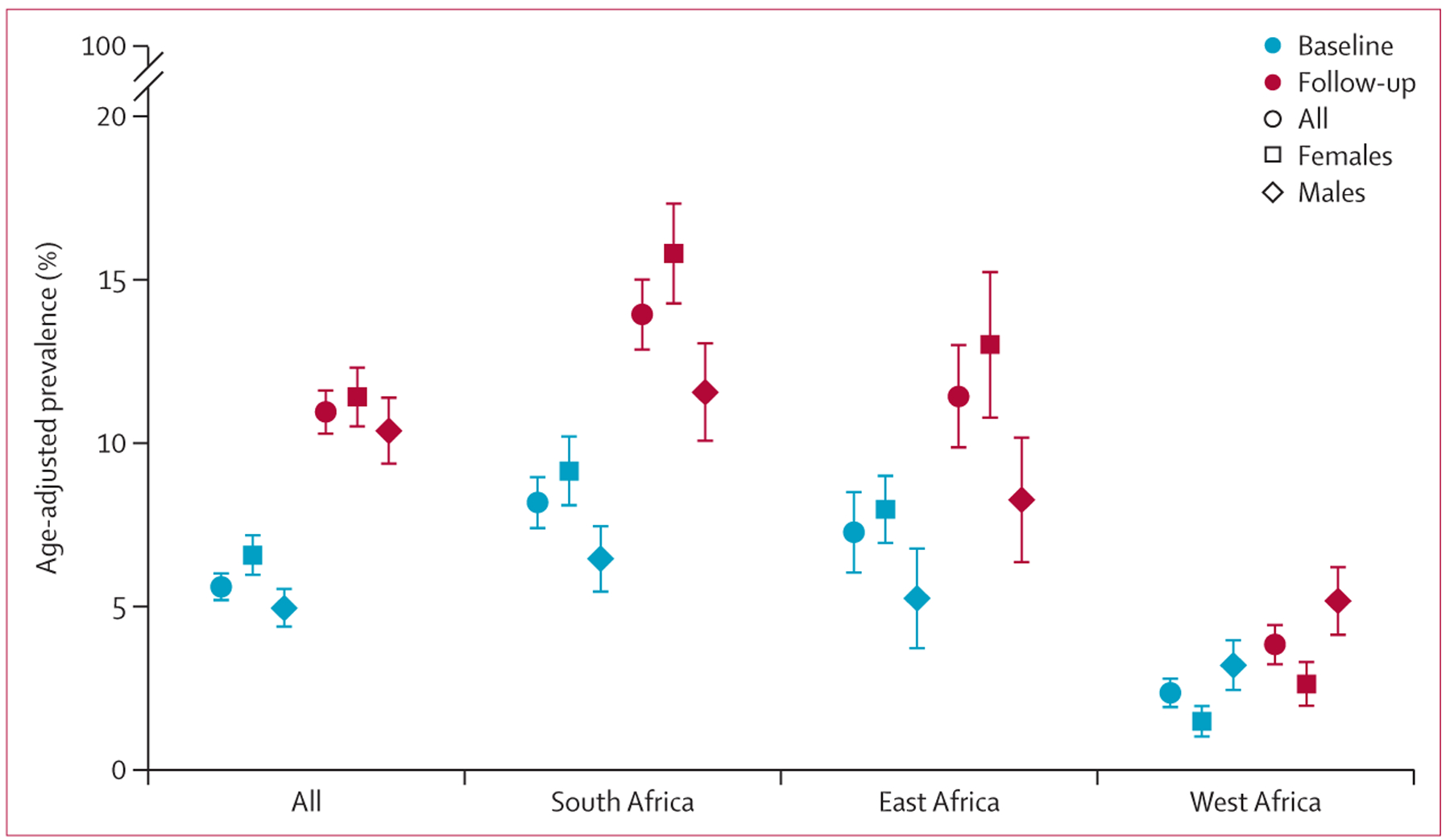
Age-adjusted prevalence of type 2 diabetes at the two study visits, overall, and stratified by study region and sex 95% CIs are shown as error bars. p<0∙0001 for males *vs* females at baseline at all sites, for females at baseline *vs* follow-up at all sites, and for males at baseline *vs* follow-up at all sites. p=0∙0013 for males *vs* females at follow-up at all sites.

The age-adjusted prevalence of type 2 diabetes was higher at follow-up regardless of the study region or sex of the participants, with a doubling of the age-adjusted prevalence for the whole sample from 5∙6% (95% CI 5∙1–6∙0) to 10∙9% (10∙2–11∙6; figure 1). The highest increase occurred in South Africa and the lowest in west Africa. In South Africa and east Africa, females exhibited a higher prevalence of type 2 diabetes compared with males at both timepoints. Conversely, in west Africa, the prevalence was higher among males than females, a pattern consistent across both visits (figure 1).

Waist and hip circumference, BMI, insulin resistance, systolic and diastolic blood pressure, total cholesterol, triglycerides, LDL cholesterol, and glucose were significantly higher at baseline among participants who developed type 2 diabetes compared with those who did not (table 2). 136 participants with incident diabetes were identified by a health-care professional, whereas 261 were diagnosed based only on glucose measurements. The only statistical difference between the two groups was in the follow-up glucose levels, ie, 5∙55 mmol/l (5∙06–6∙06) in those diagnosed by a health-care professional and 7∙78 mmol/l (7∙29–9∙32) in those identified by glucose measurements alone (p<0∙0001).

Baseline physical activity and estimated glomerular filtration rate were lower in those who developed type 2 diabetes compared with those who did not (table 2). Baseline homoeostatic model assessment for insulin resistance levels was higher in those who developed type 2 diabetes than in those who did not. Increases in waist and hip circumference were greater in those who did not develop type 2 diabetes compared with those who did. Triglycerides, glucose, and homoeostatic model assessment for insulin resistance increased more in those who developed type 2 diabetes compared with those who did not (table 2). 73 (31∙5%) of 232 participants with baseline impaired fasting glycaemia progressed to type 2 diabetes at the follow-up visit, with the highest incidence in South Africa and the lowest in west Africa (appendix p 13).

The age of enrolment among those who developed type 2 diabetes was similar across the three study regions, while participants from west Africa had lower baseline BMI at 20∙9 kg/m² (19∙0–25∙0), compared with those from east Africa at 28∙6 kg/m² (25∙1–32∙1) and South Africa at 29∙9 kg/m² (26∙8–33∙8), with similar patterns at follow-up (appendix p 14).

**Figure 2:**
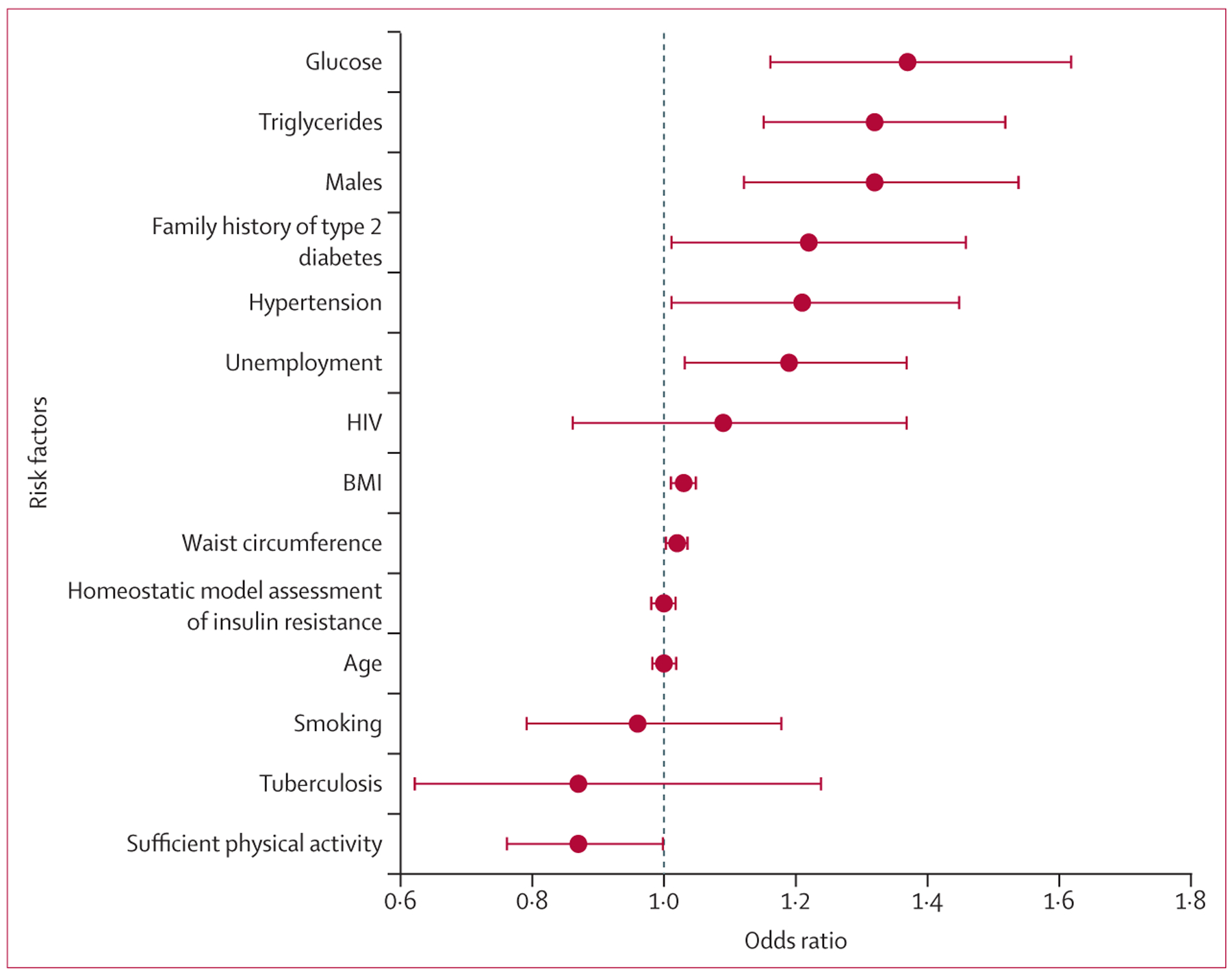
Two-stage individual participant data meta-analysis showing the association between baseline risk factors and incident type 2 diabetes Overall estimates are expressed as odds ratios with corresponding 95% CIs, depicted by circles and bars, respectively.

As shown in figure 2, there were significant associations between incident diabetes and waist circumference (adjusted odds ratio 1∙02, 95% CI 1∙01–1∙03), BMI (1∙03, 1∙02–1∙04), glucose (1∙33, 1∙22–1∙45), triglycerides (1∙32, 1∙15–1∙52), unemployment status (1∙19, 1∙03–1∙37), male sex (1∙30, 1∙11–1∙52), hypertension (1∙18 [1∙07–1∙31]), family history of type 2 diabetes (1∙21 [1∙01–1∙46]), and insufficient physical activity (0∙87 [0∙76–1∙00]). Centre-specific logistic regression models are presented in the appendix (pp 28–32), and the combined sample model (appendix pp 15–18). Predictive intervals to evaluate the effects of heterogeneity are also presented in the appendix (pp 19–23). Furthermore, we found no significant association between baseline HIV status, HIV tuberculosis co-infection, or antiretroviral therapy use and the incidence of type 2 diabetes in high HIV-prevalent study centres in east Africa and South Africa (appendix pp 24–26).

## Discussion

Our findings show a high incidence of type 2 diabetes of 14∙6 cases per 1000 person-years (95% CI 13∙4–16∙0) in a middle-aged sub-Saharan African population. We also highlight regional differences, with the highest incidence observed in South Africa, and the lowest in west Africa, possibly due to differences in obesity prevalence and sociodemographic factors, such as urbanicity. Additionally, we show that various factors at baseline including BMI, waist circumference, triglyceride and glucose levels, male sex, insufficient physical activity, unemployment, hypertension, and a family history of type 2 diabetes are associated with a greater risk of developing type 2 diabetes, with regional contrasts in the association of sex and diabetes incidence likely reflecting regional sex differences in obesity prevalence.

Longitudinal studies in middle-aged, normoglycaemic sub-Saharan African populations, with sample sizes between 256 participants and 2029 participants,^8–11^ have reported type 2 diabetes incidence ranging between four cases and 33 cases per 1000 person-years, comparable to our incidence range of six cases and 22 cases per 1000 person-years. However, our incidence of six cases per 1000 person-years in west Africa contrasts sharply with a Ghanaian study, which reported a much higher incidence rate of 33 cases per 1000 person-years (95% CI 18–57).^8^ In that study, incidence was determined in individuals living with HIV, with higher incidence in those taking combination antiretroviral drugs at baseline than in those who were antiretroviral naive. We did not replicate this association, a finding supported by a previous systematic review of seven sub-Saharan African studies.^18^ Unlike our participants from west Africa who were rural residents with a median BMI of 20∙9 kg/m^²^ (19∙0–25∙0), most of the individuals in the Ghanaian study were urban residents and had a higher mean baseline BMI of approximately 26∙0 kg/m^²^, a factor associated with increased diabetes incidence. Additionally, incident diabetes in this Ghanaian study was assessed using a glycosylated haemoglobin threshold of 6∙5%, which remains of debatable diagnostic use in African-ancestry populations.^19,20^ Our findings indicate that the peri-urban study centres, Agincourt and DIMAMO (South Africa), recorded the highest incidence of type 2 diabetes. The data suggest that these centres are undergoing a rapid epidemiological transition, influenced by urbanisation. Peri-urban settings often face a blend of health challenges seen in both rural and urban areas, which exacerbates health risks.^21^ Previous research from DIMAMO revealed a high prevalence of chronic diseases and cardiovascular risks, whereas an earlier study from Agincourt highlighted only some access to health care over the previous year, potentially elevating type 2 diabetes risk.^22,23^

An incidence of 29 cases per 1000 person-years (95% CI 15–43) was reported in 807 participants from the Democratic Republic of the Congo,^8,9^ located in central Africa, which was not represented in our study. There is insufficient information to compare all baseline characteristics of these study participants with those in our work, but some risk factors for incident type 2 diabetes were common, including glucose level and physical inactivity. A Mauritian cohort reported a type 2 diabetes incidence of 9∙8 per 1000 person-years, lower than the east African and South African centres, which are its closest geographical neighbours.^11^ Data in this study were obtained between 1987 and 1998 and might not reflect the epidemiological transition in sub-Saharan Africa in recent years. Additionally, the multi-ethnic population that included Mauritians of Creole, Chinese, and Indian ethnicity is unlikely to reflect continental Africa. A Soweto sub-cohort from the AWI-Gen study reported a type 2 diabetes incidence of 4∙4% in males and 10∙7% in females,^7^ substantially lower than the rates of 9∙6% in males and 14∙7% in females in our combined South African cohort and suggesting the high incidence in our peri-urban South African centres. Type 2 diabetes incidence in participants with baseline impaired fasting glycaemia was slightly higher (31∙5%) in this study than in a Malawian study, in which 26∙0% of these individuals developed type 2 diabetes.^24^

Our findings contrast with those from a meta-analysis of 10 893 middle-aged participants of African ancestry from the USA, enrolled in studies between the 1970s and 1980s. In that analysis, the type 2 diabetes incidence was 14·6%, over a mean follow-up of 8∙9 years,^24^ higher than our overall incidence of 8∙1%. This difference could reflect higher adiposity in the African American population or the longer follow-up duration. Our findings are however similar to those in other emerging economies, with studies from India reporting comparable type 2 diabetes incidence between 13 cases and 25 cases per 1000 person years.^25^

Diabetes was diagnosed at lower BMIs in the west African centres compared with the east and South African centres. There is a growing volume of literature on type 2 diabetes in lean phenotypes,^26,27^ which challenges BMI cutoffs of 25 kg/m^²^ in screening recommendations.^28^ Studies are increasingly showing that individuals with a normal or even low BMI could still develop type 2 diabetes.^26,27^ In a multi-site study from the USA, lean diabetes was more prominent among individuals from minority racial and ethnic groups than in non-Hispanic Whites.^29^ Evidence also suggests that multiple factors including muscle mass, low β-cell insulin output, genetics, and epigenetics could be involved in the pathophysiology of lean diabetes.^26,27^ We did not find any differences in age at diagnosis between the study regions, possibly because we only focused on a narrow baseline age group range of 40–60 years. However, a WHO STEPS pooled analysis of 56 health surveys, primarily from Africa and the Western Pacific, reported that the average age at diabetes diagnosis was 45∙0 years (95% CI 43∙8–46∙1) for females and 45∙1 years (44∙0–46∙1) for males.^30^

Our study provides further evidence for the roles of previously reported risk factors such as baseline glucose,^31^ triglycerides,^32^ male sex,^6^ measures of adiposity,^33^ physical inactivity,^34^ social determinants of health such as unemployment,^35^ family history of diabetes,^31^ and comorbid hypertension^36^ in the development of type 2 diabetes. Although we did not show an independent association between homoeostatic model assessment for insulin resistance and diabetes onset, participants who developed type 2 diabetes had a higher homoeostatic model assessment for insulin resistance level both at baseline and follow-up and had higher increases in homoeostatic model assessment for insulin resistance between the timepoints. A cross-sectional study among Black South Africans reported lower insulin sensitivity and reduced β cell function in males compared with females.^37^ In our study, the effect of sex was independent of adiposity and other variables, as the sex × BMI interaction was not significant, warranting further physiological investigation.

Our observation that participants who developed type 2 diabetes had smaller increases in waist and hip circumference than participants who did not develop type 2 diabetes also merits further comment. This finding could be due to greater catabolism in those with incident diabetes who had sub-optimal glucose control, lifestyle modification in response to a diagnosis of diabetes, or higher levels of insulin resistance, which is associated with increased visceral fat deposition but no substantial changes to waist and hip circumference. Furthermore, individuals who later developed diabetes had higher waist and hip circumference at baseline and therefore less potential to increase these measurements than those who did not develop type 2 diabetes. The increase in physical activity among participants with newly diagnosed type 2 diabetes could similarly be due to lifestyle modifications because of their diagnosis.

Our study has several strengths. We had a large sample size of more than 6500 individuals, with a follow-up duration of 5–6 years. Participants were recruited from multiple centres, across an urbanicity gradient, in different sub-Saharan African regions. Data collection was performed by centrally trained study personnel, using standardised protocols, across the six different centres and all biochemical testing was performed in a single laboratory, reducing inter-assay variability. We did however have some limitations. We recognise the significant variability in our regional incidence estimates and have therefore presented these alongside our overall incidence estimate. There is ample precedence for deriving single region-wide measures^3^ and our study has the advantage of harmonised data collection and diabetes assessment. This study did not measure social determinants of health such as access to care, health literacy, and social support that could have influenced incident diabetes. Additionally, we only used one biochemical test to diagnose diabetes, and therefore could have missed individuals who met glycosylated haemoglobin or oral glucose tolerance test diagnostic criteria, thus underestimating type 2 diabetes incidence. We also did not confirm a diagnosis of diabetes with a second test and could have incorrectly allocated diabetes status given the intraindividual variability in blood glucose. Our approach of a single test of glycaemia is, however, standard in epidemiological studies in contrast to clinical assessment of diabetes status, which requires confirmation of abnormal results in the absence of diabetes-related symptoms.^14,38^ Factors such as tuberculosis were self-reported and could have been underreported, introducing reporting bias. We also assumed all cases of incident diabetes were due to type 2 diabetes, which is the most likely form given the demographics of our study population. Despite its limitations, our study makes a valuable contribution to the literature as, to the best of our knowledge, the largest study to date on the incidence of diabetes in sub-Saharan Africa. Our findings have major implications for public health, with a doubling of type 2 diabetes prevalence during our study period in all centres. Targeted interventions are urgently needed in sub-Saharan Africa to address type 2 diabetes risk factors and reduce its incidence, while health-care systems must be restructured to identify and manage individuals with type 2 diabetes and ameliorate its social and economic impact.

## Supplementary Material

1

## Figures and Tables

**Table 1:** Incidence of type 2 diabetes in all participants and stratified by study regions and sex

	Cases per 1000 person-years (95% CI)			p value
	All	Females (n=3629)	Males (n=2736)	
All (n=6365)	14∙6 (13∙4–16∙0)	15∙3 (13∙7–17∙2)	13∙6 (11∙8–15∙7)	>0∙99
South Africa (n=2674)	21∙8 (19∙5–24∙4)*	24∙2 (21∙2–27∙7)*	18∙0 (14∙7–21∙9)*	<0∙0001
East Africa (n=1081)	19∙5 (16∙1–23∙8)*	19∙5 (15∙1–25∙3)*	19∙6 (14∙5–26∙5)*	>0∙99
West Africa (n=2610)	5∙5 (4∙4–6∙9)	3∙6 (2∙5–5∙2)	7∙9 (5∙9–10∙4)	<0∙0001

* p<0·001 *vs* west Africa. Group comparisons were calculated using the a Poisson regression and p values were obtained after Bonferroni correction, with a p<0∙05 level of significance.

**Table 2:** Baseline levels and changes in measurements in study participants who did and did not develop type 2 diabetes over the follow-up period

	Baseline measurements			Changes between baseline and follow-up		
	Developed type 2 diabetes at follow-up (n=489)	Did not develop type 2 diabetes at follow-up (n=5406)	p value	Developed type 2 diabetes at follow-up (n=489)	Did not develop type 2 diabetes at follow-up (n=5406)	p value
Age, years	50∙4 (5∙6)	49∙8 (5∙7)	0∙15	5∙7 (1∙1)	5∙5 (1∙0)	0∙92
BMI, kg/m^²^	30∙0 (24∙7 to 35∙1)	22∙7 (20∙0 to 27∙5)	<0∙0001	−0∙01 (−1∙71 to 1∙55)	0∙08 (–1∙10 to 1∙35)	>0∙99
Waist, cm	97∙8 (15∙6)	84∙0 (13∙9)	<0∙0001	0∙93 (10∙02)	3∙03 (8∙28)	<0∙0001
Hip, cm	107∙7 (16∙4)	96∙2 (14∙2)	<0∙0001	–0∙07 (9∙42)	2∙04 (7∙87)	<0∙0001
Waist:hip ratio	0∙92 (0∙34)	0∙87 (0∙16)	0∙0025	0∙01 (−0∙03 to 0∙05)	0∙01 (−0∙02 to 0∙05)	>0∙99
Systolic blood pressure, mm Hg	128∙3 (116∙0 to 141∙5)	120∙0 (108∙0 to 134∙5)	0∙0012	6∙5 (–9∙0 to 20∙5)	4∙5 (−7∙0 to 15∙5)	0∙30
Diastolic blood pressure, mm Hg	83∙8 (13∙2)	78∙6 (13∙1)	0∙0022	0∙53 (13∙.15)	0∙88 (11∙42)	>0∙99
Physical activity, min per week	720 (180 to 2100)	1200 (330 to 2880)	<0∙0001	70 (−510 to 1035)	0 (−1050 to 920)	<0∙0001
Total cholesterol, mmol/L	4∙26 (1∙13)	3∙82 (1∙11)	0∙0002	0∙73 (1∙17)	0∙66 (0∙99)	>0∙99
Triglycerides, mmol/L	0∙98 (0∙72 to 1∙35)	0∙74 (0∙54 to 1∙06)	<0∙0001	0∙27 (–0∙04 to 0∙69)	0∙18 (−0∙04 to 0∙42)	<0∙0001
HDL cholesterol, mmol/L	1∙09 (0∙91 to 1∙30)	1∙13 (0∙93 to 1∙39)	0∙15	0∙19 (0∙39)	0∙17 (0∙43)	>0∙99
LDL cholesterol, mmol/L	2∙61 (0∙94)	2∙25 (0∙88)	0∙0014	0∙35 (0∙96)	0∙38 (0∙78)	>0∙99
Glucose, mmol/L	5∙20 (4∙70 to 4∙85)	4∙79 (4∙38 to 5∙21)	<0∙0001	2∙33 (1∙12 to 4∙10)	0∙53 (0∙02 to 1∙00)	<0∙0001
Glucose levels in those with impaired fasting glucose (ie, between 6∙1 mmol/L and 6∙9 mmol/L), mmol/L	6∙39 (6∙22 to 6∙60)	6∙30 (6∙17 to 6∙52)	>0∙99	1∙63 (0∙88 to 4∙71)	−0∙68 (−1∙14 to –0∙17)	<0∙0001
Homoeostatic model assessment for insulin resistance	1∙50 (0∙64 to 2∙91)	0∙83 (0∙37 to 2∙01)	<0∙0001	1∙77 (–0∙04 to 5∙78)	0∙05 (−0.64 to 0.70)	<0∙0001
Estimated glomerular filtration rate, ml per min⁻¹ (1∙73 m)⁻²	99∙4 (84∙7 to 107∙1)	101∙2 (89∙6 to 108∙3)	0∙0001	−8∙2 (−17∙3 to −9∙7)	−9∙6 (−19.7 to 1.0)	0∙30

Data are mean (SD) or median (IQR). Type 2 diabetes p values obtained after Bonferroni correction with a <0∙05 level of significance.

## Research in context

### Evidence before this study

On July 10, 2024, we did a PubMed search for articles on the incidence of type 2 diabetes in sub-Saharan Africa. We used the search terms “incidence” AND “diabetes mellitus” AND “sub-Saharan African countries (as defined by the 2023 World Bank list)” with no limitations on language or publication year. Our search identified 17 studies from 12 countries, which were highly heterogeneous, with incidence ranging from 4 to 63 cases per 1000 person-years, follow-up periods from 1 to 11 years, and sample sizes ranging from 243 to 56 298 study participants. Only four of these studies focused on the middle-aged population (ie, those aged 40–60 years) and these were conducted in single countries. We extended the search to include articles investigating the risk factors associated with incident type 2 diabetes in sub-Saharan Africa by incorporating the additional search term “risk factors” into the original query. This search identified 100 articles of which only four were relevant. Baseline risk factors including age, physical inactivity, impaired glucose tolerance, metabolic syndrome, high leptin levels in men, and antiretroviral exposure were associated with incident type 2 diabetes. However, these studies were conducted in single countries, sample sizes varied between 256 and 2029, and participants were taking older antiretroviral drug regimens. Participants in three of these studies were recruited between 1987 and 2007 and might therefore not reflect the epidemiological transition under way in sub-Saharan Africa in the past 15 years.

### Added value of this study

This study is the largest prospective cohort study in sub-Saharan Africa to collect harmonised data on incident type 2 diabetes and its risk factors in middle-aged adults from south, east, and west sub-Saharan African countries. Our data were collected from multiple study centres, encompassing diverse demographic strata and ranging from rural subsistence farmers to more urbanised communities at different stages of the epidemiological transition. Our study revealed differences in the incidence of type 2 diabetes among the three sub-Saharan Africa regions, with the highest incidence observed in South Africa with 21∙8 cases (95% CI 19∙5–24∙4) per 1000 person-years, followed by east Africa with 19∙5 cases (16∙1–23∙8) per 1000 person-years, and the lowest in west Africa with 5∙5 cases (4∙4–6∙9) per 1000 person-years. Furthermore, we observed moderate-to-high heterogeneity in the associations of age, triglycerides levels, glucose levels, and hypertension with increased risk of developing type 2 diabetes, with the associations more significant at the study centres with a higher incidence of type 2 diabetes. We also showed that the prevalence of type 2 diabetes nearly doubled within approximately six years of follow-up, underscoring the rapid progression and growing burden of type 2 diabetes in the region.

### Implications of all the available evidence

The available evidence shows that the incidence of type 2 diabetes in sub-Saharan Africa is high, particularly in urban communities and countries at later stages of the epidemiological transition. Context-specific strategies are necessary to mitigate the risk of developing diabetes. Implementing such strategies will not only improve health outcomes but also reduce the burden on poorly resourced regional health-care systems.
