# Supplementary material for: Incident type 2 diabetes and its risk factors in men and women aged 40–60 years from four sub-Saharan African countries: results from the AWI-Gen study
Source: Lancet Glob Health. Author manuscript; Available in PMC 2025 May 15. (PMC12079105; doi:10.1016/S2214-109X(24)00520-5)
Supplement: 1 [file NIHMS2061326-supplement-1.pdf]

THE LANCET
Global Health

Supplementary appendix

This appendix formed part of the original submission and has been peer reviewed. We post it as supplied by the authors.

Supplement to: Chikwati RP, Crowther NJ, Ramsay M, et al. Incident type 2 diabetes and its risk factors in men and women aged 40–60 years from four sub-Saharan African countries: results from the AWI-Gen study. *Lancet Glob Health* 2025; **13:** e459–66.

Table of Contents

Supplementary Table 1: Comparison of baseline factors between participants who were retained in the cohort and those who were lost to follow-up 6

Supplementary Table 2: Comparison of baseline factors, prevalence and incidence of diabetes across the study centres 8

Supplementary Table 3A: Regional comparison of T2DM incidence rate ratios 10

Supplementary Table 3B: Regional comparison of T2DM incidence rate ratios between men and women 10

Supplementary Table 4: Baseline levels and changes in measurements in women who did and did not develop diabetes after follow-up 11

Supplementary Table 5: Baseline levels and changes in measurements in men who did and did not develop diabetes after follow-up 12

Supplementary Table 6: Participants with baseline impaired fasting glucose levels who developed diabetes at the follow-up visit 13

Supplementary Table 7A: Baseline age and BMI levels in newly diagnosed participants with diabetes 14

Supplementary Table 7B: Follow-up age and BMI levels in newly diagnosed participants with diabetes 14

Supplementary Table 8: Baseline factors associated with incident diabetes in all study centres 15

Supplementary Table 9: Multi-level hierarchical models showing baseline factors associated with incident diabetes in all study centres 17

Supplementary Table 10: Leave-one-out analysis and predictive intervals on the association baseline glucose levels and incident type 2 diabetes mellitus 19

Supplementary Table 11: Leave-one-out analysis and predictive intervals on the association baseline triglyceride levels and incident type 2 diabetes mellitus 20

Supplementary Table 12: Leave-one-out analysis and predictive intervals on the association baseline age and incident type 2 diabetes mellitus 21

Supplementary Table 13: Leave-one-out analysis and predictive intervals on the association baseline hypertension and incident type 2 diabetes mellitus 22

Supplementary Table 14: Leave-one-out analysis and predictive intervals on the association baseline BMI and incident type 2 diabetes mellitus 23

Supplementary Table 15: Association of HIV-TB co-infection and HIV mono-infection with incident diabetes in Nairobi, DIMAMO and Agincourt (n=1,800) 24

Supplementary Table 16: Association of HIV and ART exposure with incident diabetes in East and South African centres (n=3,481) 25

Supplementary Table 17: Association of ART exposure with incident diabetes in individuals living with HIV in East and South African centres (n=708) 26

Supplementary Figure 1: Flowchart showing the selection of study participants.. 27

Supplementary Figure 2: Forest plots displaying 2-stage individual participant data meta-analyses results for the association of baseline physical activity, employment and family history of diabetes mellitus with incident type 2 diabetes mellitus. 28

Supplementary Figure 3: Forest plots displaying 2-stage individual participant data meta-analyses results for the association of sex and insulin resistance with incident type 2 diabetes mellitus. 29

Supplementary Figure 4: Forest plots displaying 2-stage individual participant data meta-analyses results for the association of baseline body mass index (BMI) and waist circumference with incident type 2 diabetes mellitus. 30

Supplementary Figure 5: Forest plots displaying 2-stage individual participant data meta-analyses results for the association of baseline age, hypertension, fasting glucose and triglycerides with incident type 2 diabetes mellitus. 31

Supplementary Text

Sampling frames

Participants were selected using random sampling methods based on existing population sample frames from each study site. The unit of random selection was individuals at the study centres. The AWI-Gen sample size was based on detecting an allele frequency of 0·04 for cardiometabolic risk factors, such as blood pressure. At least 12,000 participants was required to achieve over 94% power (α = 0·05) to detect a genetic effect size of 1·2.[1] The target enrolment was 12,000 participants aged 40–60, with an approximately equal number of men and women (2,000 participants per study site). The site-specific approaches used are outlined below:

Nanoro

The sampling frame included 6,090 individuals aged 40–60, with a higher proportion of women [4,067 (67·0%)] than men [2,023 (33·0%)]. To address this imbalance, the sampling frame was adapted on a village-by-village basis to include equal numbers of randomly selected women and men. Participants meeting eligibility criteria were enrolled, resulting in 2,097 individuals enrolled at baseline, with a participation rate of 34·4%.

Navrongo

The sampling frame included 2,400 individuals. Approximately 20·0% more individuals were sampled at baseline to account for non-response, refusals, migration, and deaths. The geographical region showed an uneven distribution of the two main ethnolinguistic groups (Kassena and Nankana). To address this, participants were purposefully selected to achieve roughly equal proportions of these groups and a balanced ratio of men to women. A total of 2,016 participants were ultimately enrolled into the cohort, yielding a participation rate of 84·0%.

Nairobi

The sampling frame included 2,349 individuals. Participants were purposefully selected using a geographical sampling frame that covered the two peri-urban districts of Korogocho and Viwandani, until roughly equal numbers of women and men had been recruited from each district. A total of 2003 (85·0%) individuals accepted to participate and were then enrolled into the cohort.

DIMAMO

A total of 5479 individuals aged 40–60 years were identified from the HDSS site database, participants were selected randomly from the identified individuals. In the end, 1,399 (70·0%) were eligible and consented to participate.

Agincourt

Approximately 2,000 randomly selected individuals aged 40–60 were invited to participate, with stratification by sex to achieve equal numbers of women and men. Since the area's permanent population is biased toward women, a gender-specific sampling approach was used to ensure balance. Of those invited, 1,465 (73·0%) were successfully recruited. Additionally, 1,021 individuals over the age of 60, overlapping with participants from the Health and Aging in Africa: A Longitudinal Study of an INDEPTH Community in South Africa (HAALSI),[2] were recruited to bring the total enrolment to 2,486 individuals.

In Agincourt, the individuals invited to participate were a subset of participants from the Health and Aging in Africa: A Longitudinal Study of an INDEPTH Community (HAALSI). The sampling frame included 3,891 individuals, of whom 3,220 were aged 40-60 years, and an additional 671 were over 60 years of age. A total of 2,486 participants were enrolled in the cohort (64·0% participation rate), comprising 2,039 individuals aged 40-60 years and 447 individuals over 60 years.

Soweto

While complex, the sampling was also purposeful. A total of 2,031 participants were enrolled at baseline. Approximately 702 women from the 'Study of Women Entering and Endocrine Transition' (SWEET) were enrolled into AWI-Gen.[3] An additional 300 women and 1,000 men were then recruited through random selection using a geographic sampling frame covering the Soweto region.

Study variables

Body mass index (BMI) was calculated by dividing weight in kilograms by the square of height in meters. Socioeconomic status was determined based on a cumulative score of household assets, which was then categorised into quintiles. Employment status was categorised as either currently employed or unemployed. Moderate-vigorous intensity physical activity (MVPA) was assessed using the Global Physical Activity Questionnaire (GPAQ)[15] and considered sufficient if ≥150 minutes per week, and insufficient if <150 minutes per week.[16] Current smoking and alcohol consumption status were classified as either "yes" or "no". A self-reported diagnosis of tuberculosis within the last 12 months by a healthcare practitioner was used to assess tuberculosis status. HIV status was defined by either self-report or by a voluntary government-approved rapid HIV test which was only offered at the study centres in South Africa and Kenya. Amongst individuals living with HIV, the use of antiretroviral therapy was self-reported. At the West African study centres where HIV testing was not offered, negative status was assigned for all participants who did not know their HIV status, thus aligning with the low national prevalence levels during recruitment (below 1%) (2013–2017). Diabetes treatment was not used as part of the definition of a diagnosis of diabetes, as we assumed a diagnosis of diabetes would be a prerequisite for treatment.

Biochemical assays

All biochemical assays were conducted at the same laboratory using a Randox Plus clinical chemistry analyser (Randox Laboratories Ltd, Crumlin, UK). for glucose (measuring range 0·36–35·00 mmol/l), triglycerides (measuring range 0·13–5·60 mmol/L), total cholesterol (measuring range 0·30–17·20 mmol/L), high-density lipoprotein cholesterol (HDL-C) (measuring range 0·05–3·80 mmol/l), and creatinine (measuring range 11·80–24·48 µmol/l). The coefficient of variation for all assays was less than 2·3%. Low-density lipoprotein cholesterol (LDL-C) was calculated using the Friedewald formula.[4] Insulin levels were measured using the Immulite 1000 chemistry analyser (Siemens, Berlin, Germany) (measuring range 2–300 µIU/ml). Insulin resistance was calculated using the homeostatic model assessment of insulin resistance (HOMA-IR).[5] The estimated glomerular filtration rate (eGFR) was calculated using the Chronic Kidney Disease-Epidemiology Collaboration (CKD-EPI) equation without the African American ethnicity correction formula.[6]

1. Ramsay M, Crowther N, Tambo E, Agongo G, Baloyi V, Dikotope S, et al. H3Africa AWI-Gen Collaborative Centre: a resource to study the interplay between genomic and environmental risk factors for cardiometabolic diseases in four sub-Saharan African countries. Glob Health Epidemiol. 2016;1:e20.

2. Gómez-Olivé FX, Montana L, Wagner RG, Kabudula CW, Rohr JK, Kahn K, et al. Cohort Profile: Health and Ageing in Africa: A Longitudinal Study of an INDEPTH Community in South Africa (HAALSI). International Journal of Epidemiology. 2018 Jun 1;47(3):689–690j.

3. Jaff NG, Norris SA, Snyman T, Toman M, Crowther NJ. Body composition in the Study of Women Entering and in Endocrine Transition (SWEET): A perspective of African women who have a high prevalence of obesity and HIV infection. Metabolism. 2015 Sep;64(9):1031–41.

4. Friedewald WT, Levy RI, Fredrickson DS. Estimation of the Concentration of Low-Density Lipoprotein Cholesterol in Plasma, Without Use of the Preparative Ultracentrifuge.

5. Matthews DR, Hosker JR, Rudenski AS, Naylor BA, Treacher DF, Turner RC, et al. Homeostasis model assessment: insulin resistance and fl-cell function from fasting plasma glucose and insulin concentrations in man.

6. Levey AS, Stevens LA, Schmid CH, Iii AFC, Feldman HI, Kusek JW, et al. A New Equation to Estimate Glomerular Filtration Rate. 2009;

Logistic regression

Collinearity between independent variables was assessed using the variance inflation factor, with a threshold set at <5. Waist circumference and BMI were collinear and were therefore analysed in separate models. A third model, adjusted for baseline measures of T2DM risk factors, including baseline BMI, was run to investigate the relationship between absolute change in BMI between baseline and follow-up visits and the incidence of T2DM. Logistic regression models were run firstly for all six centres combined and then for five centres combined, with Soweto omitted as family history of diabetes, alcohol consumption and history of tuberculosis were not collected there. We performed sensitivity analyses using multilevel models to account for possible clustering of samples by study centres.

We chose two-stage over one-stage individual participant data meta-analyses because some covariates, included family history of diabetes, alcohol consumption, and history of tuberculosis were not available from all centres.

Sensitivity analyses

12·6% (95% CI: 11·3–14·0) of participants in South Africa developed diabetes compared to 9% (95% CI: 8·2–11·9) in East Africa, and 3·1% (95% CI: 2·5–3·8) in West Africa. In the total population, incident T2DM occurred in 489 (8·1%) individuals, with 298 (8·9%) women and 191 (7·3%) men developing diabetes.

At baseline, random blood glucose measurements were collected from 129 individuals (2·0%), and from 150 individuals (2·4%) at the follow-up visit. Based solely on these measurements, 2 individuals (1·6%) were classified as having diabetes at baseline, compared to 7 individuals (4·7%) at the follow-up visit.

We compared follow up glucose levels and changes in BMI and waist and hip circumference between those with incident diabetes who were diagnosed at the follow-up visit based on glucose levels and those who were diagnosed by a healthcare professional during the follow-up period to assess the impact of probable therapy on these variables. Those diagnosed by a healthcare professional had glucose levels of 5·55 mmol/l (5·06 – 6·06 with a BMI change 0·07 kg/m^2 (-1·55 – 1·46), waist circumference change of -0·19 ± 9·02 cm and a hip circumference change of -0·44 ± 9·43 cm. Participants with incident diabetes identified by glucose measurements alone had glucose levels of 7·78 mmol/l (7·29 – 9·32), with a BMI change 0·23 (-1·36 – 1·79), waist circumference change of 2·02 ± 10·02 cm and a hip circumference change of 0·93 ± 9·20 cm at follow-up.

In sex stratified analyses, similar findings to the combined cohort were observed (Supplementary Tables 4 and 5) except for higher baseline HDL-C levels in men who did not develop T2DM compared to those who did and a longer follow-up period and older age in women who developed diabetes compared to those that did not. In addition, eGFR decreased significantly more in women who did not develop diabetes.

In Supplementary Table 9, sensitivity analyses waist circumference was included instead of BMI. Waist circumference was associated with incident T2DM (adjusted OR 1·02; 95% CI, [1·01–1·03)] with similar associations between other baseline factors and incident T2DM as in the model that included BMI (data not shown). When participant data from Soweto were excluded, most of the significant associations remained except for sufficient physical activity and employment status which were no longer associated with lower odds of incident T2DM (Supplementary Table 8). Including family history of diabetes mellitus showed a significant association with incident T2DM (1·54 [1·05–2·26]). In the model adjusted for baseline measures of T2DM risk factors, including baseline BMI, change in BMI was not associated with incident T2DM. Similarly, change in waist circumference was not associated with incident DM after adjustment for baseline waist circumference. A gender x BMI interaction term was added to each of the regression models and was found to be non-significant in all (p-values ranged from 0·10 to 0·65).

The one-stage logistic regression showed significant associations were observed between incident T2DM and baseline BMI (adjusted OR 1·09 [95% CI 1·07–1·11]), glucose (2·00 [1·66–2·40]), triglycerides (1·77 [1·49–2·10]), unemployment status (1·41 [1·10–1·82]), men (1·48 [1·09–2·00]), hypertension (1·58 [1·23–2·02]) and sufficient physical activity 0·72 [0·54–0·96]) (Supplementary Table 8). Sensitivity analyses, which included waist circumference instead of BMI and excluded the Soweto study centre, are presented in Supplementary Tables 8 and 9.

Analyses of the associations of HIV and tuberculosis and incident T2DM

Univariate and multivariable logistic regression models combining the four centres in East and South Africa where HIV and tuberculosis were prevalent, were constructed to analyse the associations of HIV, tuberculosis, HIV-tuberculosis co-infection and antiretroviral therapy use with incident T2DM. A two-stage individual participant data meta-analysis, combining logistic regression analyses on the association of HIV and tuberculosis with incident T2DM conducted in each of the East and South African centres, was performed.

The baseline prevalence of HIV in the combined cohort was 12·9 (95% CI: 12·1–13·8)%, higher in East [Nairobi, 14·3 (12·2–16·6)%, and South Africa [Agincourt 38·2 (34·8–41·6)%; DIMAMO 19·6 (16·3–23·3)%; Soweto 20·0 (17·7–22·6)%] but lower in West Africa [Nanoro 0·5 (0·2–1·0)% and Navrongo 0·7 (0·4–1·4)%]. In total, 708 individuals were living with HIV, and 563 had ever taken antiretroviral therapy of whom 410 (72·8%) were currently taking antiretroviral therapy. Self-reported tuberculosis exposure was 4·9 (4·3–5·5)% in the combined cohort and higher in East Africa [Nairobi, 10·4 (8·7–12·3)%], and South Africa [Agincourt, 9·9 (8·0–12·2)% and DIMAMO, 6·0 (4·2–8·4)%] but lower in West Africa [Nanoro, 1·1 (0·7–1·2)% and Navrongo, 0·6 (0·3–1·3)%]. The crude prevalence of HIV-tuberculosis co-infection was 2·2 (1·8–2·7)%. In the meta-analysis of data from the four centres in East and South Africa with high HIV prevalence, baseline HIV status was not significantly associated with the incidence of diabetes (OR 1·09; 95% CI, [0·86–1·37)] (Supplementary Figure 6). Furthermore, the risk of incident diabetes in individuals with a previous history of tuberculosis at these centres did not differ from the unexposed group (OR 0·87; 95% CI, [0·62–1·24)] (Supplementary Figure 6). In logistic regression analyses of data from the four centres, there were no significant associations between either HIV-tuberculosis co-infection or HIV-mono-infection with the incidence of diabetes (Supplementary Table 15). Baseline antiretroviral use was not associated with the incidence of diabetes in participants from East and South Africa (OR 0·87; 95% CI, [0·61–1·40)] (Supplementary Table 16). Further analyses which only included individuals living with HIV also demonstrated no significant association of antiretroviral exposure with the incidence of diabetes (OR 0·65; 95% CI, [0·32–1·35)] (Supplementary Table 17).

Assessment of heterogeneity in meta-analyses

The level of significance for heterogeneity using the I^2 index was categorised as low (< 25%), moderate (25–75 %), and high (>75 %). The threshold for determining heterogeneity using the Cochrane Q test was set at a significance level of 0·01. In addition, the dispersion of the τ^2 indices were used to complement the assessment for heterogeneity. In meta-analyses with moderate and high heterogeneity, we used the leave-one out internal validation to assess the robustness of the pooled estimates. This was done by sequentially excluding one study centre at a time and recalculating the meta-analytic estimates. Furthermore, we calculated the predictive interval for each meta-analysis.

We observed moderate to high heterogeneity in the 2-stage individual participant data meta-analyses on the association between baseline glucose, age, hypertension, triglycerides and BMI and incident T2DM (Supplementary Figures 4 and 5). We then performed leave-one-out analyses and presented the predictive intervals on each of these variables in Supplementary Tables 10–14. The results were consistent with the associations from the overall meta-analyses for glucose, triglycerides, and BMI. However, we observed null associations from the respective predictive intervals, except when glucose values from DIMAMO (1·04–2·02) and triglyceride values from Navrongo were excluded (1·02–1·56). BMI maintained positive associations in both the leave-one-out analyses and the predictive interval analyses. Baseline age was only positively associated with developing T2DM in the analyses that excluded DIMAMO (Supplementary Table 12). Baseline hypertension was only positively associated with developing T2DM in the analyses that excluded Agincourt (Supplementary Table 14).

Supplementary Table 1: Comparison of baseline factors between participants who were retained in the cohort and those who were lost to follow-up

Variables	Total sample size (n=10,596)	Retained in cohort (n=6553; 61·8%)	Lost to follow-up (n=4,043; 38·2%)	*p* value
Age, years	50·0 ± 5·8	49·9 ± 5·7	50·0 ± 5·9	>0·99
Women, n (%)	5,843 (55·1)	3,756 (57·3)	2,087 (51·6)	0·0004
Region, n (%):				
South Africa	4,622 (43·6)	2,763 (42·2)	1,859 (46·0)	<0·0001
East Africa	1,883 (17·8)	1,100 (16·8)	783 (19·3)	
West Africa	4,091 (38·6)	2,690 (41·0)	1,401 (34·7)	
Education, n (%):				
No formal education	3,775 (36·4)	2,425 (38·1)	1,350 (34·0)	<0·0001
Primary	3,394 (32·8)	2,055 (32·2)	1,339 (33·7)	
Secondary	2,828 (27·3)	1,687 (26·4)	1,141 (28·7)	
Tertiary	363 (3·5)	216 (3·4)	147 (3·7)	
Employment status:				
Employed	7,214 (68·7)	4,589 (70·5)	2,625 (65·7)	0·0006
SES, n (%):				
First	1,540 (14·8)	868 (13·4)	672 (16·8)	<0·0001
Second	2,269 (21·8)	1,394 (21·6)	875 (21·9)	
Third	1,873 (18·0)	1,163 (18·1)	710 (17·8)	
Fourth	2,176 (20·9)	1,353 (21·0)	823 (20·6)	
Fifth	2,575 (24·7)	1,662 (25·8)	913 (22·9)	
Physical activity, min/week	1050 (300–2625)	1080 (300–2700)	1020 (300–2520)	0·54
Alcohol consumption:				
Yes	3,577 (41·7)	2,187 (42·4)	1,390 (40·6)	>0·99
No	5,009 (58·3)	2,975 (57·6)	2,034 (59·4)	
Smoking, n (%)				
Yes	2,862 (27·1)	1,618 (24·7)	1,244 (30·9)	0·0007
No	7,710 (72·9)	4,925 (75·3)	2,785 (69·1)	
SBP, mm Hg	121·5 (109·0–136·5)	121·0 (109·0–135·5)	122·0 (109·5–137·5)	0·11
DBP, mm Hg	78·0 (70·0–87·5)	78·0 (70·0–87·5)	78·5 (70·5–88·0)	0·54
BMI, kg/m^2	23·2 (20·1–28·5)	23·4 (20·2–28·9)	22·9 (19·9–28·0)	0·0005
Waist, cm	85·4 ± 14·8	85·8 ± 14·8	84·8 ± 14·6	0·03
Hip, cm	94·1 (86·6–105·2)	94·8 (87·0–106·0)	93·5 (86·0–104·0)	0·03
WHR	0·87 ± 0·07	0·88 ± 0·07	0·88 ± 0·07	>0·99
TC, mmol/L	3·85 ± 1·13	3·86 ± 1·13	3·84 ± 1·14	>0·99
TGs, mmol/L	0·77 (0·56–1·10)	0·77 (0·56–1·10)	0·78 (0·57–1·10)	>0·99
HDL-C, mmol/L	1·13 (0·93–1·38)	1·13 (0·93–1·37)	1·13 (0·93–1·37)	>0·99
LDL-C, mmol/L	2·28 ± 0·90	2·28 ± 0·89	2·26 ± 0·91	>0·99
Glucose, mmol/L	4·82 (4·39–5·31)	4·84 (4·40–5·31)	4·79 (4·35–5·31)	>0·99
HOMA-IR	0·92 (0·38–2·22)	0·92 (0·38–2·23)	0·92 (0·38–2·17)	>0·99
eGFR, ml min^{-1} [1·73 m]$^{-2}$	101·0 (88·6–108·1)	100·9 (88·8–108·0)	101·0 (88·1–108·2)	>0·99
Family history of diabetes:				
Yes	803 (9·6)	459 (9·1)	344 (10·3)	>0·99
No	7,570 (90·4)	4,583 (90·9)	2,987 (89·7)	
Diabetes mellitus, n (%)	603 (5·8)	344 (5·4)	259 (6·5)	0·27
Hypertension, n (%)	3,642 (34·4)	2,190 (33·4)	1,452 (35·9)	0·27

Living with HIV, n (%)	1325 (12·5)	769 (11·7)	556 (13·8)	0·08
TB in past 12 months, n (%)	103 (1·1)	47 (0·8)	56 (1·5)	0·03

Data presented as mean ± standard deviation or median (interquartile range) or counts (percentage); SES-socioeconomic status (quintiles), SBP-systolic blood pressure, DBP-diastolic blood pressure, WHR-waist-hip-ratio, TC-total cholesterol, TGs-triglycerides, HDL-C-high density lipoprotein cholesterol, LDL-C-low density lipoprotein cholesterol, HOMA-IR- homeostatic model assessment for insulin resistance, TB-tuberculosis, p-values obtained after Bonferroni correction, level of significance $p < 0{\cdot}05$. Crude prevalence calculated for diabetes, hypertension, HIV and TB.

Supplementary Table 2: Comparison of baseline factors, prevalence and incidence of diabetes across the study centres

Risk factors	All Sites (n=10,596)	Nanoro, West Africa (n=2,085)	Navrongo, West Africa (n=2,006)	DIMAMO, South Africa (n=1,162)	Agincourt, South Africa (n=1,459)	Nairobi, East Africa (n=1,883)	Soweto, South Africa (n=2,001)	*p* value
Urban-rural status	-	Rural	Rural	Peri-urban	Peri-urban	Urban	Urban	..
Age, years	50·0 ± 5·8	49·8 ± 5·8	51·1 ± 5·7	50·4 ± 6·0	50·8 ± 5·8	48·7 ± 5·3	49·3 ± 5·8	<0·0001
Women	5,843 (55·1)	1,040 (49·9)	1,089 (54·3)	808 (69·5)	889 (60·9)	1,023 (54·3)	994 (49·7)	0·0003
BMI, kg/m²	23·2 (20·1 – 28·5)	20·4 (18·6 – 22·6)	21·0 (19·3 – 23·1)	26·9 (21·1 – 33·1)	26·1 (22·1 – 33·3)	24·4 (21·1 – 28·5)	28·5 (23·0–33·9)	<0·0001
Waist circumference, cm	82·0 (74·0 – 95·0)	76·7 (72·5 – 83·1)	73·4 (69·0 – 79·0)	89·0 (76·4 – 100·4)	90·0 (80·0 – 101·0)	85·8 (77·3 – 95·3)	94·0 (82·2 –104·0)	<0·0001
Current Smoking	1,763 (16·7)	142 (6·8)	408 (20·4)	248 (21·4)	157 (10·8)	228 (12·1)	580 (29·2)	<0·0001
Current Alcohol Consumption	3,577 (36·2)	1,322 (63·5)	1,297 (64·8)	318 (27·4)	289 (19·8)	135 (18·7)	*	<0·0001
Sufficient MVPA	8,808 (83·1)	1,680 (80·6)	1,710 (85·2)	1,119 (96·3)	1,144 (78·4)	1,747 (92·8)	1,408 (70·4)	<0·0001
Diabetes prevalence	5·7 (5·2–6·1]	3·4 (2·7–4·3)	1·1 (0·7–1·7)	9·0 (7·5–10·8)	6·2 (5·1–7·6)	6·7 (5·6–7·9)	9·3 (8·2–10·7)	<0·0001
Urban-rural status diabetes prevalence	-	2·3 (1·9–2·8)	2·3 (1·9–2·8)	7·5 (6·5–8·6)	7·5 (6·5–8·6)	8·1 (7·2–9·0)	8·1 (7·2–9·0)	<0.0001
T2DM incidence per 1000	14·6 (13·4–16·0)	8·3 (6·5–10·6)	2·4 (1·5–3·9)	25·9 (20·5–32·8)	22·7 (18·5–27·8)	19·6 (16·1–23·8)	19·9 (17·0–23·3)	<0·0001
Urban-rural status T2DM incidence per 1000	-	5·5 (4·4–6·9)	5·5 (4·4–6·9)	23·9 (20·5–27·9)	23·9 (20·5–27·9)	19·7 (17·4–22·4)	19·7 (17·4–22·4)	<0·0001
Obesity	2,179 (20·6)	37 (1·8)	57 (2·8)	426 (36·7)	455 (31·2)	367 (19·4)	837 (41·8)	<0·0001
Hypertension	3,642 (34·4)	336 (16·1)	487 (24·3)	504 (43·4)	757 (51·9)	493 (26·2)	1,065 (53·2)	<0·0001
Hypertriglyceridaemia	1,023 (9·7)	103 (4·9)	49 (2·4)	163 (14·0)	167 (11·5)	223 (11·8)	318 (15·9)	<0·0001
Hypercholesterolaemia	1,449 (13·7)	131 (6·3)	62 (3·1)	189 (16·3)	246 (16·9)	357 (19·0)	464 (23·2)	<0·0001
Living with HIV	12·9 (12·1–13·8)	0·5 (0·2–1·0)	0·7 (0·4–1·4)	19·6 (16·3–23·3)	38·2 (34·8–41·6)	14·3 (12·2–16·6)	20·0 (17·7–22·6)	<0·0001
TB exposure in past 12 months	4·9 (4·3–5·5)	1·1 (0·7–1·2)	0·6 (0·3–1·3)	6·0 (4·2–8·4)	9·9 (8·0–12·2)	10·4 (8·7–12·3)	*	<0·0001

Data presented as mean ± standard deviation or numbers (percentage). Data on crude prevalence is expressed as percentage (95% confidence intervals), MVPA: moderate-vigorous intensity physical activity. *Data on alcohol consumption and TB exposure were missing for Soweto. Significance testing across the sites completed by one-way ANOVA or chi squared test. Definitions were as follows; Sufficient MVPA: ≥150 min/week, obesity: BMI ≥30 kg/m²,

hypertension: systolic blood pressure ≥140 mmHg and/or diastolic blood pressure ≥90 mmHg, diabetes mellitus: fasting plasma glucose ≥ 7·0 mmol/L and/or confirmed diagnosis by a health professional, hypertriglyceridaemia: triglycerides ≥1·69 mmol/L and hypercholesterolaemia: cholesterol ≥ 5·18 mmol/L.

Supplementary Table 3A: Regional comparison of T2DM incidence rate ratios

	IRR	95% CI	*p* value
South vs East Africa	1·34	0·38–1·43	0·26
South vs West Africa	4·10	3·19–5·26	<0·0001
East vs West Africa	3·60	2·66–4·86	<0·0001

Supplementary Table 3B: Regional comparison of T2DM incidence rate ratios between men and women

	IRR	95% CI	*p* value
Overall	1·02	0·82–1·20	>0·99
South Africa	0·65	0·52–0·83	<0.0001
West Africa	1·50	1·20–1·90	<0.0001
East Africa	0·89	0·38–2·00	0·99

Supplementary Table 3C: Regional comparison of T2DM incidence rate ratios between men

	IRR	95% CI	*p* value
South vs East Africa	0·87	0·61–1·26	>0·99
South vs West Africa	2·20	1·57–3·07	<0·0001
East vs West Africa	2·51	1·67–3·78	<0·0001

Supplementary Table 3D: Regional comparison of T2DM incidence rate ratios between women

	IRR	95% CI	*p* value
South vs East Africa	1·32	0·90–1·77	0·18
South vs West Africa	7·32	4·90–13·20	<0.0001
East vs West Africa	5·53	5·53–8·59	<0.0001

IRR – incidence rate ratio, Group comparisons were calculated using the Poisson regression , p-values obtained after Bonferroni correction.

Supplementary Table 4: Baseline levels and changes in measurements in women who did and did not develop diabetes after follow-up

Variables	Baseline measurements			Changes between baseline and follow-up		
	Developed T2DM at follow-up (n=298)	No T2DM at follow-up (n=3,037)	*p* value	Developed T2DM at follow-up (n=298)	No T2DM at follow-up (n=3,037)	*p* value
Age, years	50·3 ± 5·6	49·9 ± 5·6	>0·99	5·9 ± 1·2	5·7 ± 1·0	0·0006
BMI, kg/m^2	33·3 (28·6–37·9)	24·1 (20·5–30·5)	<0·0001	-0·02 (-1·91–1·61)	0·25 (-1·09–1·64)	0·45
Waist, cm	101·1 ± 15·6	85·8 ± 15·0	<0·0001	0·7 ± 10·8	3·0 ± 8·8	<0·0001
Hip, cm	113·4 ± 16·2	99·9 ± 15·8	<0·0001	-0·03 ± 10·66	2·09 ± 8·27	<0·0001
WHR	0·89 ± 0·08	0·86 ± 0·16	0·0006	0·01 ± 0·09	0·01 ± 0·07	>0·99
SBP, mm Hg	127·0 (115·5–140·5)	118·0 (105·5–133·5)	<0·0001	6·0 (-9·5–19·0)	4·5 (-7·0–15·5)	>0·99
DBP, mm Hg	83·6 ± 12·6	78·0 ± 13·2	0·0007	-0·4 ± 13·0	0·1 ± 11·3	>0·99
Physical activity	645 (145–1800)	1080 (300–2790)	<0·0001	150 (-570–1160)	0 (-1035–900)	0·30
TC, mmol/L	4·34 ± 1·18	3·82 ± 1·13	<0·0001	0·75 ± 1·12	0·69 ± 0·94	>0·99
TGs, mmol/L	1·01 (0·71–1·36)	0·75 (0·55–1·04)	<0·0001	0·27 (-0·01–0·65)	0·19 (-0·03–0·41)	<0·0001
HDL-C, mmol/L	1·10 (0·94–1·31)	1·11 (0·91–1·35)	>0·99	0·18 (0·02–0·39)	0·17 (-0·02–0·36)	>0·99
LDL-C, mmol/L	2·72 ± 0·95	2·28 ± 0·88	0·0005	0·37 ± 0·91	0·41 ± 0·73	>0·99
Fasting glucose, mmol/L	5·13 (4·65–5·70)	4·75 (4·38–5·16)	<0·0001	2·49 (1·24–4·66)	0·60 (0·14–1·02)	<0·0001
Glucose levels in those with IFG, mmol/L	6·30 (6·21–6·60)	6·33 (6·18–6·52)	>0·99	2·51 (0·75–5·11)	-0·69 (-1·01–-0·21)	<0·0001
HOMA-IR	1·59 (0·70–2·88)	0·96 (0·39–2·12)	<0·0001	2·23 (0·26–5·89)	0·07 (-0·64–0·85)	<0·0001
eGFR (ml min^{-1} [1·73 $m]^{-2}$)	95·5 (80·3–104·9)	98·5 (85·1–105·8)	0·15	-11·6 (-22·3– -2·1)	-15·2 (-26·8– -5·0)	0·0008

BMI-body mass index, WHR-waist-hip-ratio, SBP-systolic blood pressure, DBP-diastolic blood pressure, TC-total cholesterol, TGs-triglycerides, HDL-C-high-density lipoprotein cholesterol, LDL-C-low-density lipoprotein cholesterol, HOMA-IR- Homeostatic Model Assessment for Insulin Resistance, IFG- impaired fasting glucose (between 6·1 and 6·9 mmol/L), eGFR-estimated glomerular filtration rate. *p* values obtained after Bonferroni correction, level of significance p <0·05.

Supplementary Table 5: Baseline levels and changes in measurements in men who did and did not develop diabetes after follow-up

Variables	Baseline measurements			Changes between baseline and follow-up		
	Developed T2DM at follow-up (n=191)	No T2DM at follow-up (n=2,369)	*p* value	Developed T2DM at follow-up (n=186)	No T2DM at follow-up (n=2,369)	*p* value
Age, years	50·6 ± 5·7	49·6 ± 5·8	0·45	5·3 ± 0·8	5·4 ± 0·8	>0·99
BMI, kg/m^2	25·4 (21·9–29·6)	21·6 (19·6–24·5)	<0·0001	-0·01 (-1·4–1·5)	-0·1 (-1·1–1·0)	>0·99
Waist, cm	92·8 ± 14·2	81·7 ± 11·9	<0·0001	1·3 ± 8·8	3·1 ± 7·5	0·03
Hip, cm	99·3 ± 10·6	91·8 ± 9·9	<0·0001	-0·1 ± 7·1	1·9 ± 7·3	<0·0001
WHR	0·93 ± 0·07	0·89 ± 0·06	0·0005	0·02 ± 0·07	0·01 ± 0·06	>0·99
SBP, mm Hg	129·0 (116·5–143·0)	122·0 (111·0–135·0)	0·0007	7·5 (-8·0–22·5)	4·0 (-7·5–15·0)	0·15
DBP, mm Hg	84·3 ± 14·2	79·4 ± 12·8	0·0004	13·3 ± 2·1	11·5 ± 1·9	>0·99
Physical activity	720 (200–2520)	1320 (360–2880)	<0·0001	90 (-600–1500)	0 (-1290–1190)	0·45
TC, mmol/L	4·11 ± 1·05	3·81 ± 1·09	<0·0001	0·68 ± 1·23	0·61 ± 1·05	>0·99
TGs, mmol/L	0·97 (0·74–1·35)	0·73 (0·54–1·07)	0·0007	0·27 (-0·11–0·78)	0·17 (-0·06–0·43)	0·15
HDL-C, mmol/L	1·06 (0·87–1·30)	1·16 (0·96–1·43)	0·001	0·18 (-0·03–0·39)	0·13 (-0·09–0·39)	>0·99
LDL-C, mmol/L	2·44 ± 0·92	2·21 ± 0·87	0·0004	0·32 ± 1·03	0·33 ± 0·83	>0·99
Glucose, mmol/L	5·24 (4·66–6·02)	4·85 (4·37–5·26)	<0·0001	2·12 (1·02–3·18)	0·44 (-0·12–0·98)	<0·0001
Glucose levels in those with IFG, mmol/L	6·40 (6·25–6·59)	6·27 (6·17–6·51)	0·60	1·53 (0·93–3·12)	-0·67 (-1·17 – -0·10)	<0·0001
HOMA-IR	1·36 (0·49–2·92)	0·64 (0·35–1·83)	<0·0001	1·20 (-0·23–4·96)	0·02 (-0·65–0·49)	<0·0001
eGFR, ml min^{-1} [1·73 m]$^{-2}$	103·4 (95·9–109·3)	104·4 (96·4–110·8)	>0·99	-4·3 (-10·5–6·8)	-5·0 (-11·4–7·2)	>0·99

BMI-body mass index, WHR-waist-hip-ratio, SBP-systolic blood pressure, DBP-diastolic blood pressure, TC-total cholesterol, TGs-triglycerides, HDL-C-high-density lipoprotein cholesterol, LDL-C-low-density lipoprotein cholesterol, HOMA-IR- Homeostatic Model Assessment for Insulin Resistance, IFG- impaired fasting glucose (between 6·1 and 6·9 mmol/L), eGFR-estimated glomerular filtration rate, p values obtained after Bonferroni correction, level of significance p <0·05.

Supplementary Table 6: Participants with baseline impaired fasting glucose levels who developed diabetes at the follow-up visit

Regions	Impaired fasting glucose, n (%)
All	73 (31·5)
South Africa	38 (43·7)
East Africa	22 (34·4)
West Africa	13 (16·1)

South Africa-Soweto, DIMAMO and Agincourt. East Africa-Nairobi, Kenya. West Africa-Navrongo, Ghana and Nanoro, Burkina Faso

Supplementary Table 7A: Baseline age and BMI levels in newly diagnosed participants with diabetes

	All regions (n=155)	South Africa (n=73)	East Africa (n=44)	West Africa (n=38)	P value
Age, years	51·7 ± 5·6	52·7 ± 5·4	50·8 ± 5·3	50·6 ± 6·2	0·52
BMI, kgm^{-2}	27·6 (21·9 – 31·7)	29·9 (26·8 – 33·8)	28·6 (25·1 – 32·1)	20·9 (19·0 – 25·0)***+++	<0·0001

***p<0.0001 vs South Africa and +++p<0.0001 vs East Africa. BMI comparisons were calculated using the Dunn's test for non-parametric pairwise comparisons with adjustment for multiple comparisons.

Supplementary Table 7B: Follow-up age and BMI levels in newly diagnosed participants with diabetes

	All regions (n=261)	South Africa (n=152)	East Africa (n=61)	West Africa (n=48)	*P* value
Age, years	56·4 ± 5·6	56·8 ± 5·8	55·0 ± 4·7	56·8 ± 6·0	0·11
BMI, kgm^{-2}	29·2 (24·0 – 34·5)	31·7 (26·6 – 35·8)	27·7 (25·0 – 33·4)	23·1 (19·9 – 26·9)***+++	<0·0001

***p<0.0001 vs South Africa and +++p<0.0001 vs East Africa. BMI comparisons were calculated using the Dunn's test for non-parametric pairwise comparisons with adjustment for multiple comparisons.

Supplementary Table 8: Baseline factors associated with incident diabetes in all study centres

Risk factors	Unadjusted OR (95% CI)	*p* value	Adjusted Model OR (95% CI) including Soweto	*p* value	Adjusted Model OR (95% CI) excluding Soweto	*p* value
Age	1·02 (1·00–1·04)	0·01	1·01 (0·99–1·03)	0·53	1·00 (0·98–1·03)	0·78
Duration of follow-up	1·13 (1·03–1·25)	0·01	1·12 (0·98–1·28)	0·09	1·05 (0·88–1·25)	0·57
Sex:						
Women	Reference		Reference		Reference	
Men	0·82 (0·69–1·00)	0·05	1·48 (1·09–2·00)	0·01	1·96 (1·39–2·77)	<0·0001
Marital status:						
Never married	Reference		Reference		Reference	
Currently married	1·07 (0·72–1·59)	0·73	1·32 (0·81–2·15)	0·27	1·11 (0·62–1·99)	0·72
Previously married	1·40 (0·93–2·13)	0·11	1·46 (0·87–2·46)	0·15	1·45 (0·78–2·68)	0·24
Level of formal education:						
None	Reference		Reference		Reference	
Primary	2·57 (2·00–3·31)	<0·0001	1·15 (0·83–1·59)	0·40	0·89 (0·62–1·28)	0·54
Secondary	2·63 (2·02–3·41)	<0·0001	1·20 (0·85–1·72)	0·30	1·12 (0·76–1·64)	0·57
Tertiary	2·26 (1·34–3·82)	0·002	0·72 (0·36–1·47)	0·37	0·93 (0·40–2·17)	0·87
Employment status:						
Employed	Reference		Reference		Reference	
Unemployed	1·68 (1·38–2·03)	<0·0001	1·41 (1·10–1·82)	0·01	1·28 (0·94–1·74)	0·11
Socioeconomic quintiles:						
First	Reference		Reference		Reference	
Second	1·43 (1·00–2·07)	0·05	1·14 (0·72–1·83)	0·57	1·14 (0·66–1·95)	0·64
Third	1·48 (1·02–2·15)	0·04	1·34 (0·83–2·17)	0·23	1·47 (0·86–2·49)	0·16
Fourth	1·45 (1·00–2·08)	0·05	1·24 (0·78–1·97)	0·36	1·11 (0·66–1·87)	0·68
Fifth	1·78 (1·26–2·52)	0·001	1·26 (0·79–1·99)	0·33	1·42 (0·86–2·33)	0·17
Family history of diabetes:			-	-		
No	Reference		-	-	Reference	
Yes	2·57 (1·90–3·50)	<0·0001	-	-	1·54 (1·05–2·26)	0·03
Smoking:						

No	Reference		Reference		Reference	
Yes	0·58 (0·43–0·79)	0·001	0·78 (0·56–1·09)	0·14	0·77 (0·51–1·16)	0·22
Alcohol consumption:			-	-		
Never	Reference		-	-	Reference	
Yes	0·41 (0·32–0·53)	0·0031	-	-	0·77 (0·55–1·08)	0·13
Living with HIV:						
No	Reference		Reference		Reference	
Yes & taking ART	0·97 (0·39–2·43)	0·95	1·15 (0·76–1·74)	0·50	1·38 (0·86–2·21)	0·18
Yes & not taking ART	1·20 (0·84–1·72)	0·31	0·82 (0·33–2·03)	0·67	0·54 (0·11–2·59)	0·44
History of TB:			-	-		
No	Reference		-	-	Reference	
Yes	0·90 (0·58–1·40)	0·36	-	-	0·75 (0·37–1·51)	0·42
Physical activity, min/wk:						
Insufficient (<150)	Reference		Reference		Reference	
Sufficient (≥150)	0·60 (0·48–0·75)	<0·0001	0·72 (0·54–0·96)	0·03	0·76 (0·53–1·07)	0·11
BMI, kgm^{-2}	1·11 (1·09–1·12)	<0·0001	1·09 (1·07–1·11)	<0·0001	1·10 (1·08–1·12)	<0·0001
Glucose, mmol/L	2·53 (2·20–2·90)	<0·0001	2·00 (1·66–2·40)	<0·0001	1·83 (1·50–2·24)	<0·0001
Triglycerides, mmol/L	1·97 (1·72–2·26)	<0·0001	1·77 (1·49–2·10)	<0·0001	2·08 (1·75–2·48)	<0·0001
LDL-cholesterol, mmol/L	1·53 (1·38–1·68)	<0·0001	1·07 (0·93–1·23)	0·36	1·05 (0·89–1·23)	0·45
HOMA	1·00 (0·99–1·02)	<0·0001	1·00 (0·98–1·02)	0·83	1·00 (0·98–1·02)	0·75
Hypertension:						
No	Reference		Reference		Reference	
Yes	2·69 (2·23–3·24)	<0·0001	1·58 (1·23–2·02)	0·001	1·61 (1·21–2·13)	0·001

HIV-human immunodeficiency virus, ART-antiretroviral therapy, TB-tuberculosis, min/wk-minutes per week, BMI-body mass index, LDL-low-density lipoprotein, HOMA-homeostatic model assessment of insulin resistance. Univariate models for the following variables: family history of diabetes, alcohol consumption, history of TB did not include the Soweto centre. All risk factors listed were included in the multivariable models.

Supplementary Table 9: Multi-level hierarchical models showing baseline factors associated with incident diabetes in all study centres

Risk factors	Unadjusted OR (95% CI)	*p* value	Adjusted Model OR (95% CI) including Soweto	*p* value	Adjusted Model OR (95% CI) excluding Soweto	*p* value
Study centre:						
Navrongo	Reference		Reference		Reference	
Nanoro	3·14 (1·80–5·47)	<0·0001	2·51 (1·98–3·17)	<0·0001	2·26 (1·71–2·98)	<0·0001
Agincourt	9·67 (5·64–16·6)	<0·0001	3·43 (2·38–4·95)	<0·0001	2·62 (1·70–4·02)	<0·0001
DIMAMO	12·08 (6·93–21·05)	0·002	3·75 (2·37–5·92)	<0·0001	3·17 (2·00–5·03)	<0·0001
Soweto	9·37 (5·56–15·08)	<0·0001	2·42 (1·35–4·33)	0·003	-	-
Age	1·02 (1·00–1·04)	0·01	1·01 (0·98–1·03)	0·60	1·00 (0·97–1·03)	0·87
Duration of follow-up	1·13 (1·03–1·25)	0·01	1·21 (1·04–1·41)	0·01	1·13 (0·94–1·36)	0·20
Women	Reference		Reference		Reference	
Men	0·82 (0·69–1·00)	0·05	1·63 (1·16–2·30)	0·01	1·95 (1·42–2·70)	<0·0001
Never married	Reference		Reference		Reference	
Currently married	1·07 (0·72–1·59)	0·73	1·34 (1·02–1·77)	0·04	1·22 (0·80–1·85)	0.36
Previously married	1·40 (0·93–2·13)	0·11	1·57 (1·06–2·32)	0·02	1·63 (0·89–2·98)	0.11
Level of formal education:						
None	Reference		Reference		Reference	
Primary	2·57 (2·00–3·31)	<0·0001	0·92 (0·58–1·45)	0·71	0·77 (0·54–1·08)	0·13
Secondary	2·63 (2·02–3·41)	<0·0001	0·96 (0·55–1·69)	0·89	0·91 (0·52–1·60)	0·75
Tertiary	2·26 (1·34–3·82)	0·002	0·62 (0·31–1·21)	0·16	0·83 (0·37–1·87)	0·66
Employment status:						
Employed	Reference		Reference		Reference	
Unemployed	1·68 (1·38–2·03)	<0·0001	1·46 (1·18–1·81)	0·001	1·34 (0·99–1·81)	0·06
Socioeconomic quintiles:						
First	Reference		Reference		Reference	
Second	1·43 (1·00–2·07)	0·05	1·12 (0·67–1·86)	0·67	1·12 (0·56–2·27)	0·75
Third	1·48 (1·02–2·15)	0·04	1·36 (0·78–2·40)	0·28	1·52 (0·74–3·11)	0·25
Fourth	1·45 (1·00–2·08)	0·05	1·24 (0·76–2·02)	0·39	1·14 (0·64–2·03)	0·65
Fifth	1·78 (1·26–2·52)	0·001	1·29 (0·77–2·16)	0·33	1·48 (0·85–2·56)	0·17
Family history of diabetes:			-	-		

No	Reference		-	-	Reference	
Yes	2·57 (1·90–3·50)	<0·0001	-	-	1·50 (1·09–2·04)	0·01
Smoking:						
No	Reference		Reference		Reference	
Yes	0·58 (0·43–0·79)	0·001	0·82 (0·65–1·04)	0·11	0·78 (0·61–1·00)	0·05
Alcohol consumption:			-	-		
Never	Reference		-	-	Reference	
Yes	0·41 (0·32–0·53)	<0·0001	-	-	0·88 (0·75–1·03)	0·12
Living with HIV:						
No	Reference		Reference		Reference	
Yes & taking ART	0·97 (0·39–2·43)	0·95	0·98 (0·74–1·29)	0·89	1·28 (1·07–1·53)	0·01
Yes & not taking ART	1·20 (0·84–1·72)	0·31	0·79 (0·45–1·39)	0·41	0·48 (0·22–1·07)	0·07
History of TB:			-	-		
No	Reference		-	-	Reference	
Yes	0·90 (0·58–1·40)	0·36	-	-	0·69 (0·31–1·52)	0·36
Physical activity, min/wk:						
Insufficient (<150)	Reference		Reference		Reference	
Sufficient (≥150)	0·60 (0·48–0·75)	<0·0001	0·69 (0·57–0·83)	<0·0001	0·73 (0·58–0·91)	0·01
BMI, kgm^{-2}	1·11 (1·09–1·12)	<0·0001	1·08 (1·05–1·11)	<0·0001	1·09 (1·07–1·11)	<0·0001
Glucose, mmol/L	2·53 (2·20–2·90)	<0·0001	1·92 (1·26–2·95)	0·002	1·76 (1·12–2·76)	0·01
Triglycerides, mmol/L	1·97 (1·72–2·26)	<0·0001	1·69 (1·32–2·16)	<0·0001	1·95 (1·57–2·43)	<0·0001
LDL-cholesterol, mmol/L	1·53 (1·38–1·68)	<0·0001	1·04 (0·91–1·20)	0·58	1·02 (0·87–1·21)	0·78
HOMA	1·00 (0·99–1·02)	<0·0001	1·00 (0·98–1·02)	0·99	1·00 (0·98–1·01)	0·84
Hypertension:						
No	Reference		Reference		Reference	
Yes	2·69 (2·23–3·24)	<0·0001	1·62 (1·16–2·25)	0·004	1·63 (1·07–2·47)	0·02

HIV-human immunodeficiency virus, ART-antiretroviral therapy, TB-tuberculosis, min/wk-minutes per week, BMI-body mass index, LDL-low-density lipoprotein, HOMA-homeostatic model assessment of insulin resistance. Univariate models for the following variables: family history of diabetes, alcohol consumption, history of TB did not include the Soweto centre. All risk factors listed were included in the multi-level hierarchal regression (adjusted) models.

Supplementary Table 10: Leave-one-out analysis and predictive intervals on the association baseline glucose levels and incident type 2 diabetes mellitus

Meta-analysis	Odds ratio	95% CI	Predictive interval	Notes
Overall	1·37	1·16–1·62	0·94–2·01	Predictive interval widened to a null association
Excluding Agincourt	1·42	1·16–1·72	0·94–2·17	Predictive interval widened to a null association
Excluding DIMAMO	1·45	1·23–1·71	1·04–2·02	Predictive interval maintains a positive association
Excluding Nairobi	1·30	1·10–1·54	0·94–2·17	Predictive interval widened to a null association
Excluding Nanoro	1·41	1·15–1·72	0·92–2·17	Predictive interval widened to a null association
Excluding Navrongo	1·34	1·12–1·60	0·90–2·00	Predictive interval widened to a null association
Excluding Soweto	1·32	1·10–1·58	0·90–1·93	Predictive interval widened to a null association

Supplementary Table 11: Leave-one-out analysis and predictive intervals on the association baseline triglyceride levels and incident type 2 diabetes mellitus

Meta-analysis	Odds ratio	95% CI	Predictive interval	Notes
Overall	1·32	1·15–1·52	0·97–1·81	Predictive interval widened to a null association
Excluding Agincourt	1·30	1·09–1·54	0·91–1·86	Predictive interval widened to a null association
Excluding DIMAMO	1·36	1·14–1·62	0·93–1·99	Predictive interval widened to a null association
Excluding Nairobi	1·32	1·10–1·57	0·90–1·93	Predictive interval widened to a null association
Excluding Nanoro	1·37	1·15–1·63	0·95–1·98	Predictive interval widened to a null association
Excluding Navrongo	1·26	1·13–1·41	1·02–1·56	Predictive interval maintains a positive association
Excluding Soweto	1·38	1·18–1·62	0·99–1·91	Predictive interval widened to a null association

Supplementary Table 12: Leave-one-out analysis and predictive intervals on the association baseline age and incident type 2 diabetes mellitus

Meta-analysis	Odds ratio	95% CI	Predictive interval	Notes
Overall	1·00	0·99–1·02	0·97–1·04	Predictive interval maintains a null association
Excluding Agincourt	1·00	0·98–1·02	0·96–1·05	Predictive interval maintains a null association
Excluding DIMAMO	1·01	1·00–1·02	1·00–1·02	Predictive interval shows a slightly positive association
Excluding Nairobi	1·00	0·98–1·03	0·96–1·05	Predictive interval maintains a null association
Excluding Nanoro	1·00	0·98–1·02	0·97–1·03	Predictive interval maintains a null association
Excluding Navrongo	1·00	0·98–1·02	0·97–1·03	Predictive interval maintains a null association
Excluding Soweto	1·00	0·98–1·02	0.96–1·05	Predictive interval maintains a null association

Supplementary Table 13: Leave-one-out analysis and predictive intervals on the association baseline hypertension and incident type 2 diabetes mellitus

Meta-analysis	Odds ratio	95% CI	Predictive interval	Notes
Overall	1·21	1·00–1·45	0·81–1·80	Predictive interval widened to a null association
Excluding Agincourt	1·33	1·17–1·50	1·17–1·50	Predictive interval maintains a positive association
Excluding DIMAMO	1·14	0·96–1·37	0·81–1·62	Predictive interval maintains a null association
Excluding Nairobi	1·17	0·94–1·47	0·73–1·89	Predictive interval maintains a null association
Excluding Nanoro	1·19	0·95–1·50	0·73–1·94	Predictive interval maintains a null association
Excluding Navrongo	1·26	1·06–1·50	0·88–1·80	Predictive interval widened to a null association
Excluding Soweto	1·18	0·94–1·49	0·73–1·92	Predictive interval maintains a null association

Supplementary Table 14: Leave-one-out analysis and predictive intervals on the association baseline BMI and incident type 2 diabetes mellitus

Meta-analysis	Odds ratio	95% CI	Predictive interval	Notes
Overall	1·04	1·02–1·05	1·01 – 1·06	Predictive interval maintains a positive association
Excluding Agincourt	1·03	1·02 – 1·04	1·02 – 1·04	Predictive interval maintains a positive association
Excluding DIMAMO	1·04	1·02 – 1·05	1·01 – 1·06	Predictive interval maintains a positive association
Excluding Nairobi	1·04	1·02 – 1·05	1·01 – 1·06	Predictive interval maintains a positive association
Excluding Nanoro	1·03	1·02 – 1·04	1·02 – 1·04	Predictive interval maintains a positive association
Excluding Navrongo	1·03	1·02 – 1·04	1·02 – 1·04	Predictive interval maintains a positive association
Excluding Soweto	1·04	1·03 – 1·05	1·03 – 1·05	Predictive interval maintains a positive association

Supplementary Table 15: Association of HIV-TB co-infection and HIV mono-infection with incident diabetes in Nairobi, DIMAMO and Agincourt (n=1,800)

Risk factors	Unadjusted OR (95% CI)	*P* value	Adjusted Model* OR (95% CI)	*P* value
HIV-TB exposure:				
Without HIV	Reference		Reference	
HIV-TB coinfection	0·44 (0·19–1·02)	0·06	0·78 (0·31–1·99)	0·61
HIV only	0·77 (0·54–1·09)	0·15	1·10 (0·72–1·68)	0·66

*Adjusted for: age, duration of follow-up, sex, marital status, education level, employment status, socioeconomic status, family history of diabetes, smoking, alcohol intake, physical activity, body mass index, glucose, triglycerides, low-density lipoprotein cholesterol, insulin resistance and hypertension

Supplementary Table 16: Association of HIV and ART exposure with incident diabetes in East and South African centres (n=3,481)

Risk factors	Unadjusted OR (95% CI)	*P* value	Adjusted Model* OR (95% CI), without Soweto	*P* value	Adjusted Model* OR (95% CI), with Soweto	*P* value
HIV and ART exposure:						
Without HIV	Reference		Reference		Reference	
With HIV, on ART	0·68 (0·47–0·98)	0·04	1·07 (0·68–1·69)	0·77	0·92 (0·61–1·40)	0·70
With HIV, not on ART	0·58 (0·23–1·46)	0·25	0·48 (0·10–2·25)	0·35	0·76 (0·31–1·88)	0·56

*Adjusted for: age, duration of follow-up, sex, marital status, education level, employment status, socioeconomic status, family history of diabetes, smoking, alcohol intake, physical activity, body mass index, glucose, triglycerides, low-density lipoprotein-cholesterol, insulin resistance and hypertension

Supplementary Table 17: Association of ART exposure with incident diabetes in individuals living with HIV in East and South African centres (n=708)

Risk factors	Unadjusted OR (95% CI)	*P* value	Adjusted Model* OR (95% CI), without Soweto	*P* value	Adjusted Model* OR (95% CI), with Soweto	*P* value
ART exposure						
Not taking ART	Reference		Reference		Reference	
Taking ART	0·76 (0·41–1·42)	0·39	0·89 (0·38–2·06)	0·78	0·92 (0·61–1·40)	0·70

*Adjusted for: age, duration of follow-up, sex, marital status, education level, employment status, socioeconomic status, family history of diabetes, smoking, alcohol intake, physical activity, body mass index, glucose, triglycerides, low-density lipoprotein cholesterol, insulin resistance and hypertension

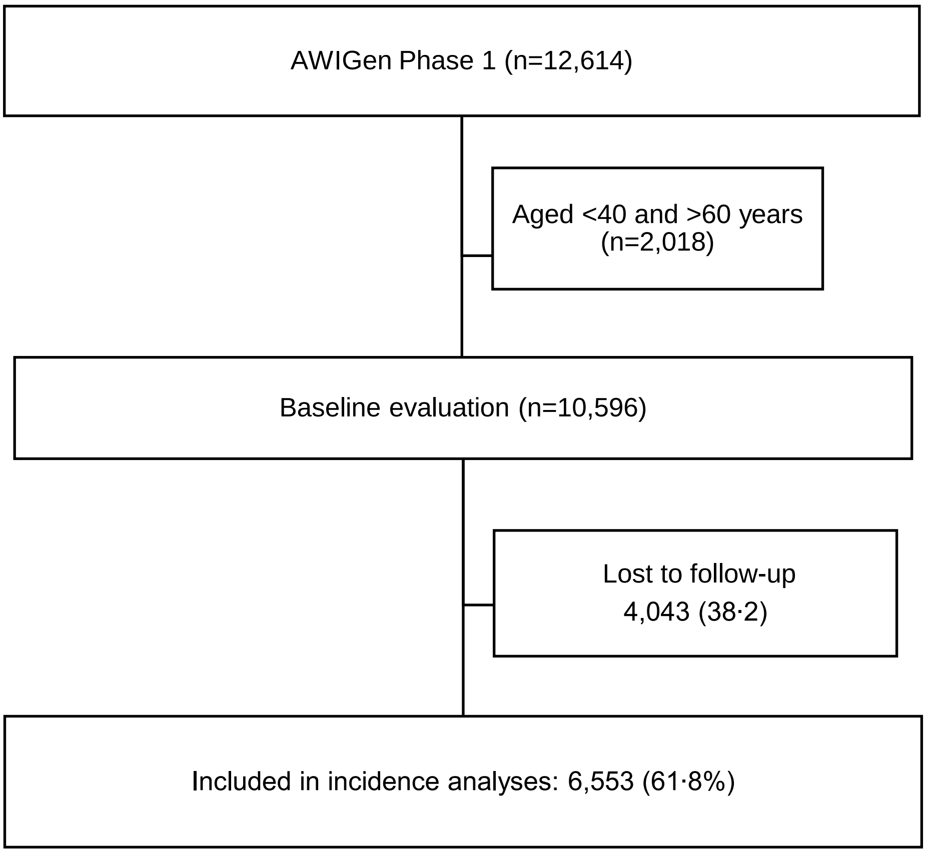

Supplementary Figure 1: Flowchart showing the selection of study participants. AWIGen- Africa-Wits International Network for the Demographic Evaluation of Populations and Their Health (INDEPTH) Partnership for Genomic studies. Out of the 4,043 participants lost to follow-up, 1,759 (43·5%) could not be contacted, 1,178 (29·1%) refused to participate, 533 (13·2%) had died, 477 (11·8%) had relocated from the study area, and 96 (2·4%) were too ill to participate.

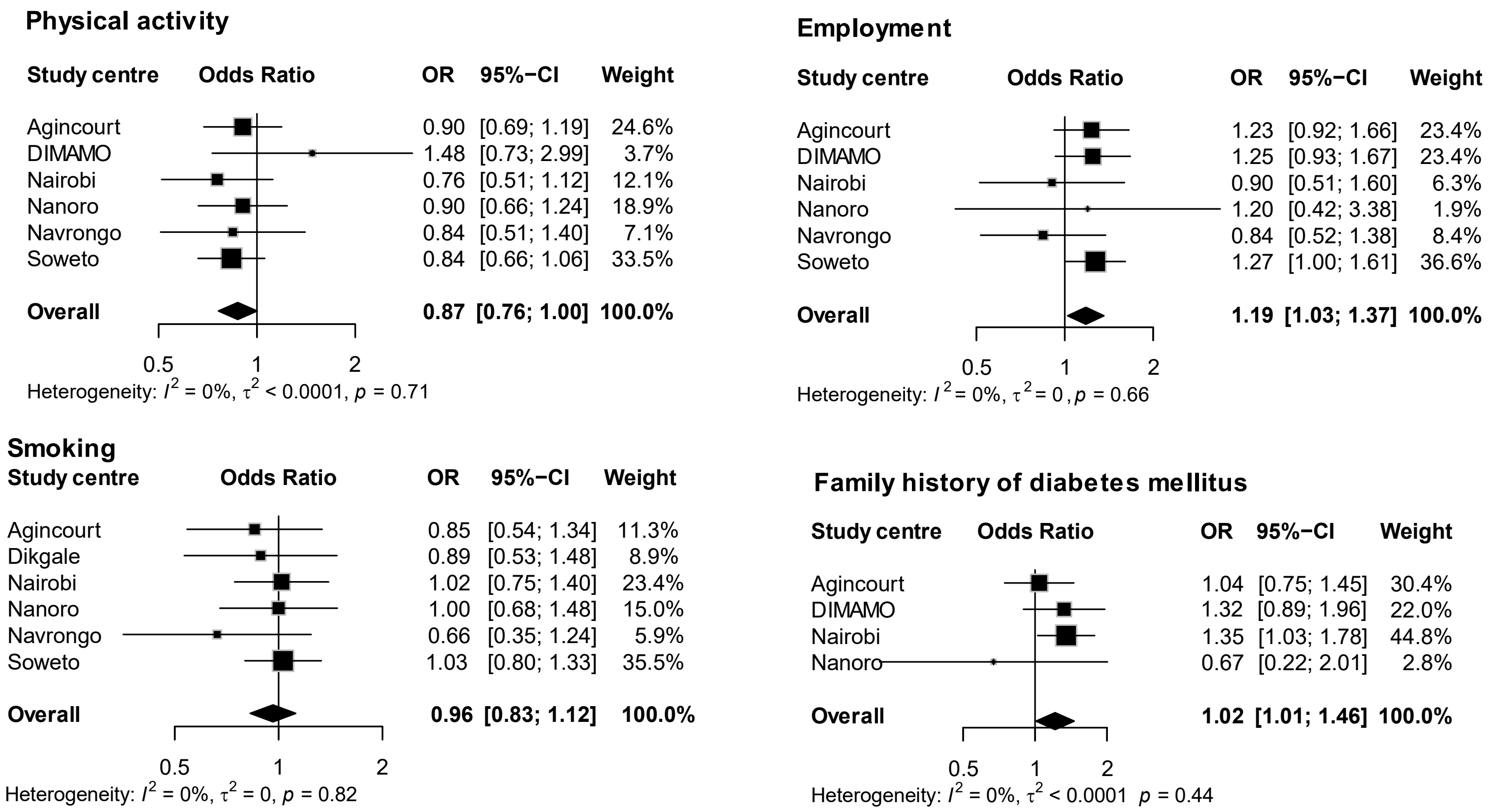

Supplementary Figure 2: Forest plots displaying 2-stage individual participant data meta-analyses results for the association of baseline physical activity, employment and family history of diabetes mellitus with incident type 2 diabetes mellitus. Overall estimates are expressed as odds ratios (OR) with corresponding 95% confidence intervals (CIs), depicted by symbols and bars, respectively. Models included age, duration of follow-up, sex, marital status, education level, employment status, socioeconomic status, family history of diabetes, smoking, alcohol intake, physical activity, HIV, TB, BMI, glucose, triglycerides, low-density lipoprotein cholesterol, insulin resistance and hypertension

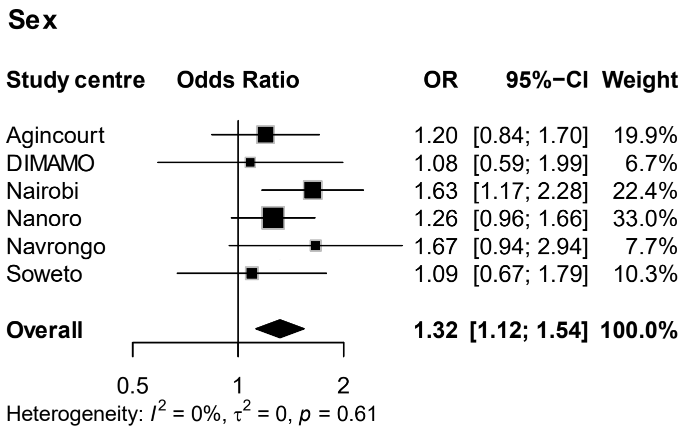

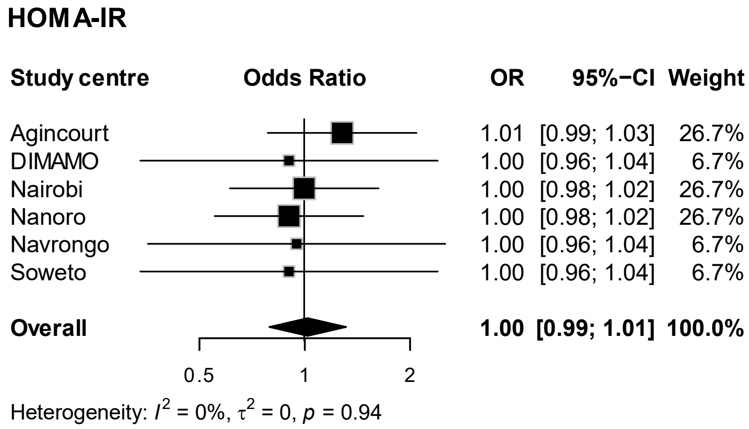

Supplementary Figure 3: Forest plots displaying 2-stage individual participant data meta-analyses results for the association of sex and insulin resistance with incident type 2 diabetes mellitus. HOMA-IR; homeostatic model assessment of insulin resistance. Overall estimates are expressed as odds ratios (OR) with corresponding 95% confidence intervals (CIs), depicted by symbols and bars, respectively. Individual site models were adjusted for : age, duration of follow-up, sex, marital status, education level, employment status, socioeconomic status, family history of diabetes, smoking, alcohol intake, physical activity, HIV, TB, body mass index, glucose, triglycerides, low-density lipoprotein cholesterol, insulin resistance and hypertension.

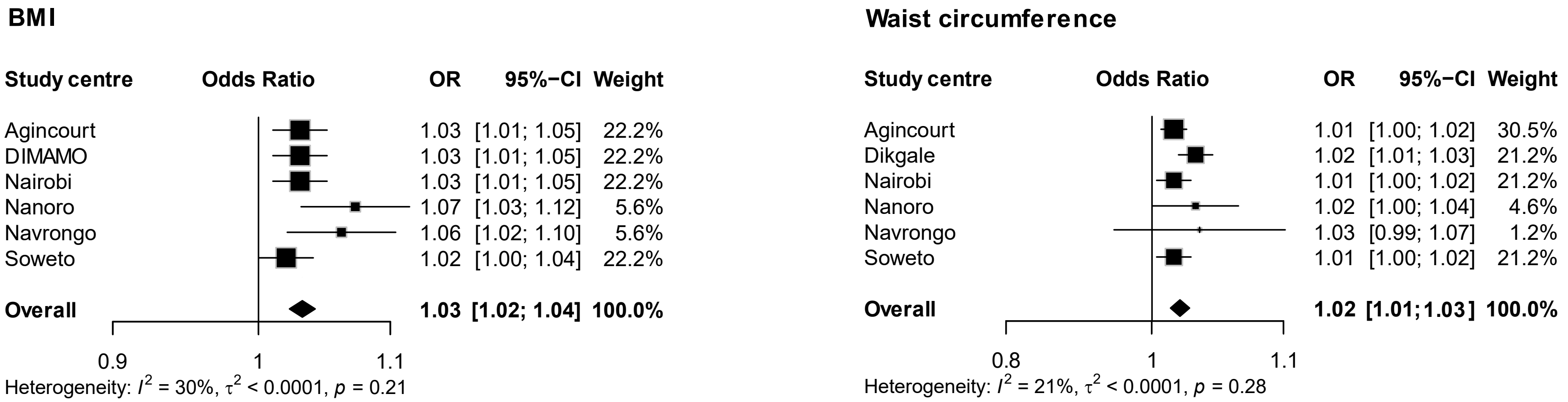

Supplementary Figure 4: Forest plots displaying 2-stage individual participant data meta-analyses results for the association of baseline body mass index (BMI) and waist circumference with incident type 2 diabetes mellitus. Overall estimates are expressed as odds ratios (OR) with corresponding 95% confidence intervals (CIs), depicted by symbols and bars, respectively. Models included age, duration of follow-up, sex, marital status, education level, employment status, socioeconomic status, family history of diabetes, smoking, alcohol intake, physical activity, HIV, TB, body mass index, glucose, triglycerides, low-density lipoprotein cholesterol, insulin resistance and hypertension.

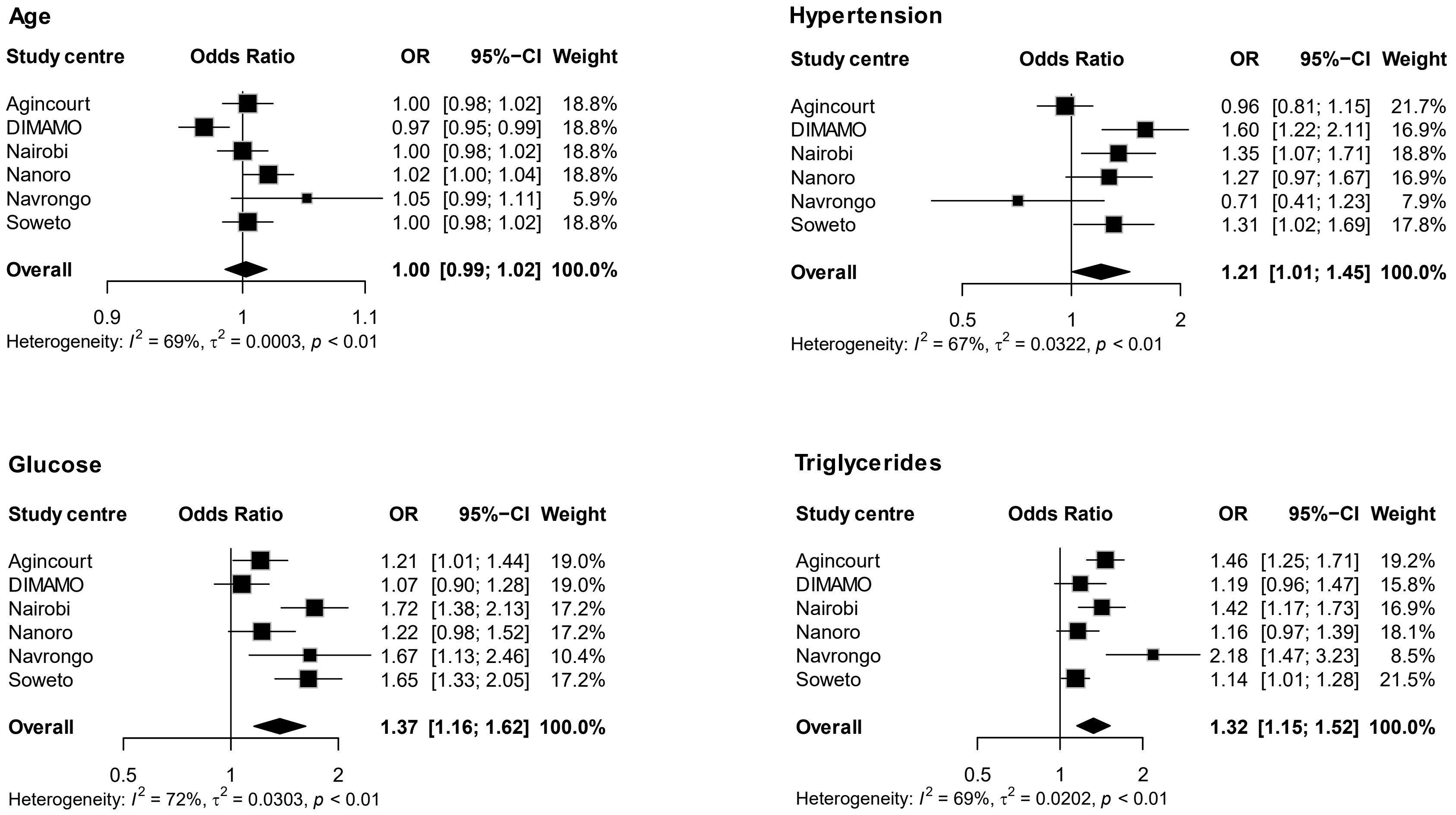

Supplementary Figure 5: Forest plots displaying 2-stage individual participant data meta-analyses results for the association of baseline age, hypertension, fasting glucose and triglycerides with incident type 2 diabetes mellitus. Overall estimates are expressed as odds ratios (OR) with corresponding 95% confidence intervals (CIs), depicted by symbols and bars, respectively. Models included age, duration of follow-up, sex, marital status, education level, employment status, socioeconomic status, family history of diabetes, smoking, alcohol intake, physical activity, HIV, TB, body mass index, glucose, triglycerides, low-density lipoprotein cholesterol, insulin resistance and hypertension.

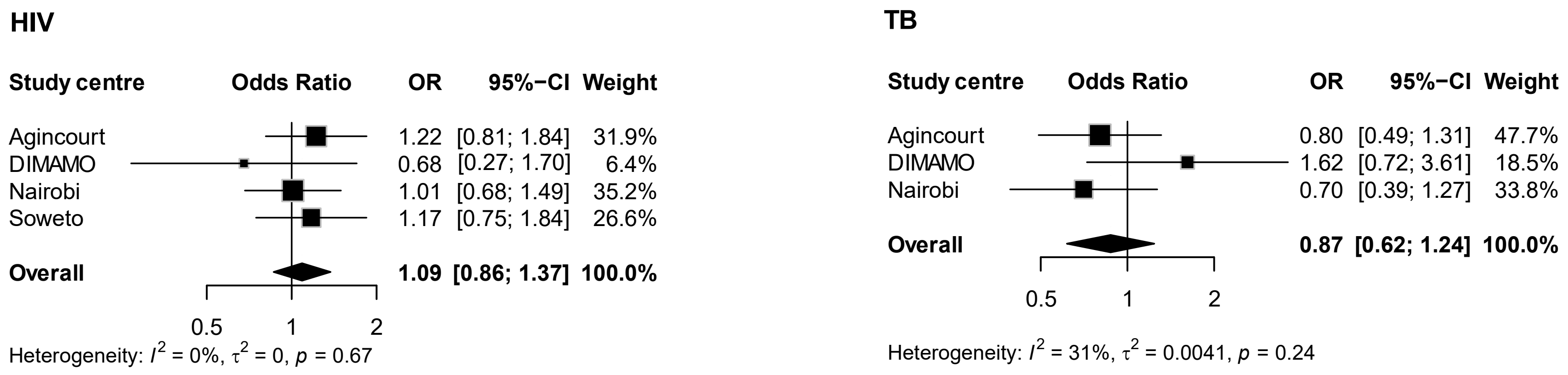

Supplementary Figure 6: Forest plots displaying 2-stage individual participant data meta-analyses results for the association of baseline HIV and tuberculosis (TB) with incident type 2 diabetes mellitus. Overall estimates are expressed as odds ratios (OR) with corresponding 95% confidence intervals (CIs), depicted by symbols and bars, respectively. Models included age, duration of follow-up, sex, marital status, education level, employment status, socioeconomic status, family history of diabetes, smoking, alcohol intake, physical activity, HIV, TB, body mass index, glucose, triglycerides, low-density lipoprotein cholesterol, insulin resistance and hypertension.
